# Genome-wide identification and functional exploration of the legume lectin genes in *Brassica napus* and their roles in *Sclerotinia* disease resistance

**DOI:** 10.3389/fpls.2022.963263

**Published:** 2022-07-22

**Authors:** Rong Zuo, Meili Xie, Feng Gao, Jie Liu, Minqiang Tang, Xiaohui Cheng, Yueying Liu, Zetao Bai, Shengyi Liu

**Affiliations:** ^1^The Key Laboratory of Biology and Genetic Improvement of Oil Crops, The Ministry of Agriculture and Rural Affairs of PRC, Oil Crops Research Institute, Chinese Academy of Agricultural Sciences, Wuhan, China; ^2^Hainan University, Haikou, China

**Keywords:** legume lectin, *Brassica napus*, *Sclerotinia sclerotiorum*, phylogenetic analysis, genome-wide association study

## Abstract

As one of the largest classes of lectins, legume lectins have a variety of desirable features such as antibacterial and insecticidal activities as well as anti-abiotic stress ability. The *Sclerotinia* disease (SD) caused by the soil-borne fungus *Sclerotinia sclerotiorum* is a devastating disease affecting most oil crops such as *Brassica napus*. Here, we identified 130 legume lectin (*LegLu*) genes in *B. napus*, which could be phylogenetically classified into seven clusters. The *BnLegLu* gene family has been significantly expanded since the whole-genome duplication (WGD) or segmental duplication. Gene structure and conserved motif analysis suggested that the *BnLegLu* genes were well conserved in each cluster. Moreover, relative to those genes only containing the legume lectin domain in cluster VI–VII, the genes in cluster I–V harbored a transmembrane domain and a kinase domain linked to the legume lectin domain in the C terminus. The expression of most *BnLegLu* genes was relatively low in various tissues. Thirty-five *BnLegLu* genes were responsive to abiotic stress, and 40 *BnLegLu* genes were strongly induced by *S. sclerotiorum*, with a most significant up-regulation of 715-fold, indicating their functional roles in SD resistance. Four *BnLegLu* genes were located in the candidate regions of genome-wide association analysis (GWAS) results which resulted from a worldwide rapeseed population consisting of 324 accessions associated with SD. Among them, the positive role of *BnLegLus-16* in SD resistance was validated by transient expression in tobacco leaves. This study provides important information on *BnLegLu* genes, particularly about their roles in SD resistance, which may help targeted functional research and genetic improvement in the breeding of *B. napus*.

## Introduction

Biotic and abiotic stresses severely disrupt the homeostasis of the plant immune system and cause great negative effects on plant growth, development, and yield (Kosová et al., 2011). Plant lectins are widely distributed in nature, and play important roles in the plant immune system (Katoch and Tripathi, 2021). Numerous induced lectins and lectin receptor-like kinases have been reported to be involved in various stress responses (Van Damme, 2022). All lectins possess at least one non-catalytic domain, which binds reversibly to a specific mono- or oligosaccharide (Nivetha et al., 2021). Legume lectins, one of the largest lectin families, are particularly abundant in legume seeds and vegetative tissues according to the early technology (Tsaneva and Van Damme, 2020). However, with the characterization of increasing legume lectins in various species, a great number of lowly expressed or induced legume lectin homologs have been discovered in non-leguminous species such as there was 54 legume lectin genes in *A. thaliana* (Lagarda-Diaz et al., 2017), suggesting their ubiquitous presence in plants. Interestingly, classical Fabaceae lectins only contain the representative legume lectin domain (250 aa), while the ubiquitous legume lectins additionally include a long (60–150 aa) extension or an extended receptor-like kinase (RLK) domain in the C terminus, such as *LegLus* in *A. thaliana* (Damme et al., 2008; Tsaneva and Van Damme, 2020; De Coninck and Van Damme, 2021). In addition, numerous classical legume lectins have been specifically characterized at the structure level (Manoj and Suguna, 2001). Among these legume lectins, ConA (concanavalin A) in *Canavalia gladiata* (Jacq.) DC. is the first lectin whose primary and 3D structure have been resolved (Edelman et al., 1972; Hardman and Ainsworth, 1972; Cavada et al., 2019). Despite of the conserved primary, secondary and tertiary structure of the legume lectin domain, its quaternary structure shows considerable variations. Most classical legume lectins can form homodimers or homo-tetramers (“dimers of dimers”; Lagarda-Diaz et al., 2017), and the monomer structure displays a jelly-roll tertiary fold like a β-sandwich (a flattened six-stranded β-sheet and a curved seven-stranded β-sheet interconnected by several loops) of 25–30 kDa, which contains a carbohydrate recognition domain (CRD) and metal binding sites for divalent cations (Ca^2+^ and Mn^2+^; Gautam et al., 2018b; Katoch and Tripathi, 2021). One loop important for carbohydrate binding is highly variable, leading to changes in carbohydrate specificity (Katoch and Tripathi, 2021). However, it remains quite difficult to study the structure of LecRLK (receptor kinases with a legume lectin domain) due to glycosylation. For instance, crystallization and preliminary x-ray studies of *AtLecRK-I.9* (*At5g60300*) failed to clearly elucidate its structure, owing to the rapid crystal decay during X-ray diffraction (Li et al., 2016).

Generally, legume lectins have vital functions in plant growth and development, including the storage of carbohydrates and hormones, and cell–cell interaction *via* binding to cell surface receptors (Lagarda-Diaz et al., 2017; Gautam et al., 2018b). In recent years, there has been increasing evidence demonstrating that a certain number of legume lectins are involved in the response to various stimuli (such as hormones and abiotic stress), and plant-insect, bacterial and fungal interactions (Bellande et al., 2017). For example, *ConA* inhibits the development of tomato moth (*Lacanobia oleracea*) and peach-potato aphid (*Myzus persicae*) when overexpressed in transgenic plants (Gatehouse et al., 1999). Besides, ConA is toxic to grain aphids through induction of gut cell apoptosis (Sprawka et al., 2015). The lectins from *Egyptian Pisum sativum* seeds could inhibit the growth of *Aspergillus flavus*, *Trichoderma viride* and *Fusarium oxysporum* (Li et al., 2012). In *P. sativum*, *PslecRLK* (pea lectin receptor-kinase) promotes the tolerance to high salinity stress by alleviating osmotic and ionic stresses and upregulating stress-responsive genes (Joshi et al., 2010), and overexpression of *PslecRLK* in rice could improve the salinity tolerance by inhibiting sodium accumulation (Passricha et al., 2019). In *Glycine max*, *GmLecRLK* contributes to salt stress tolerance by regulating salt-responsive genes in soybean (Zhang et al., 2022). In *A. thaliana*, the A4 (*LecRKA4.1/At5g01540*, *LecRKA4.2/At5g01550* and *LecRKA4.3/At5g01560*) subfamily of lectin-receptor kinases negatively regulates abscisic acid response in seed germination (Xin et al., 2009). Mechanically, the antifungal activity of legume lectins can be mainly ascribed to the indirect effect of their binding with cell wall components, which can affect fungal survival or other activities like spore germination (Leal et al., 2012). Some legume seed proteins can bind with carbohydrate components of the bacterial cell wall or extracellular glycans and prevent the entry of microorganisms into the cytoplasm (Fonseca et al., 2022). Previous studies have demonstrated that lectins from chickpea have antibacterial activity against some bacterial pathogens such as *Escherichia coli*, *Bacillus subtilis*, *Salmonella marcescens*, and *Pseudomonas syringae* (Gautam et al., 2018a). In addition, the lectins from *Egyptian P. sativum* seeds can inhibit the growth of *Aspergillus flavus*, *Trichoderma viride* and *Fusarium oxysporum* (Sitohy et al., 2007). Furthermore, some recent studies have revealed that several *lecRKs* play important roles in stress signal transduction in plant immunity (Desclos-Theveniau et al., 2012; Singh and Zimmerli, 2013). *AtLecRK-V.5* (*AT3G59700*) represses stomatal immunity induced by *P. syringae* (*P. syringae*; Arnaud et al., 2012; Desclos-Theveniau et al., 2012). *AtLecRK-IX.2* (*AT5G65600*) not only contributes to plant resistance to *P. syringae* (Luo et al., 2017), but also plays an important role in plant immunity by phosphorylating the bacterial effector *AvrPtoB* at S335 and thereby potentially decreasing its virulence (Xu et al., 2020). Moreover, AtLecRK-VI.2 (AT5G01540) is a potential primary eNAD (P)^+^-binding receptor, and plays a central role in the biological induction of SAR (systemic acquired resistance; Wang et al., 2019). AtLecRK-VI.2 associates with the pattern-recognition receptor FLS2 and primes *Nicotiana benthamiana* (*Nicotiana. L*) pattern-triggered immunity. AtLecRK-I.9 is involved in protein–protein interaction with proteins containing RGD (arginine-glycine-aspartic acid) as a ligand, and plays a structural and signaling role at the plant cell surface against pathogens (Bouwmeester et al., 2011; Huang et al., 2014; Wang et al., 2016). In addition, AtLecRK-I.9 acts as an extracellular ATP receptor in *A. thaliana* (Wang et al., 2018b), which plays an important role in systemic wound response activation (Pham et al., 2020; Myers et al., 2022). AtLecRK-I.9 also affects jasmonate signaling to contribute to resistance to *P. syringae* (Balagué et al., 2017).

*Sclerotinia* stem rot caused by *Sclerotinia sclerotiorum* is one of the devastating diseases affecting the yield and quality of oil crops, including *B. napus*. Several genes or factors contributing to plant SD resistance have been reported to date, such as *OsPGIP2* (Wang et al., 2018a), *GDSL1* (Ding et al., 2020), *BnF5H* (Cao et al., 2022), Cinnamoyl-CoA Reductase 2 (Liu et al., 2021a), and *WRKY15* and *WRKY33* (Liu et al., 2018), which have provided some insights into plant defense against *S. sclerotiorum*. However, the SD resistance of *B. napus* belongs to quantitative resistance, and thus its genetic mechanism remains largely unknown. Considering the potent antibacterial and antifungal activities of legume lectins, they may be promising targets to be utilized for the improvement of SD resistance. Here, we performed a genome-wide characterization of the legume lectin genes in *B. napus*, and then phylogenetically and structurally categorized the *BnLegLu* members. The potential contribution of *BnLegLu* genes to SD resistance was investigated based on the spatiotemporal expression profiles under abiotic stimulation and *S. sclerotiorum* induction, and the candidate genes were further screened by genome-wide association study (GWAS) with a rapeseed population comprising 324 accessions collected all over the world. A transgenic study with transient expression of *BnLegLu-16* in tobacco (*Nicotiana L*) leaves validated its positive role in regulating SD resistance under *S. sclerotiorum* inoculation. The findings of the present study may lay a solid foundation for further exploring the role of *LegLus* in rapeseed defense against SD.

## Results

### Identification and characterization of *LegLus* in *Brassica napus*

**Figure 1:**
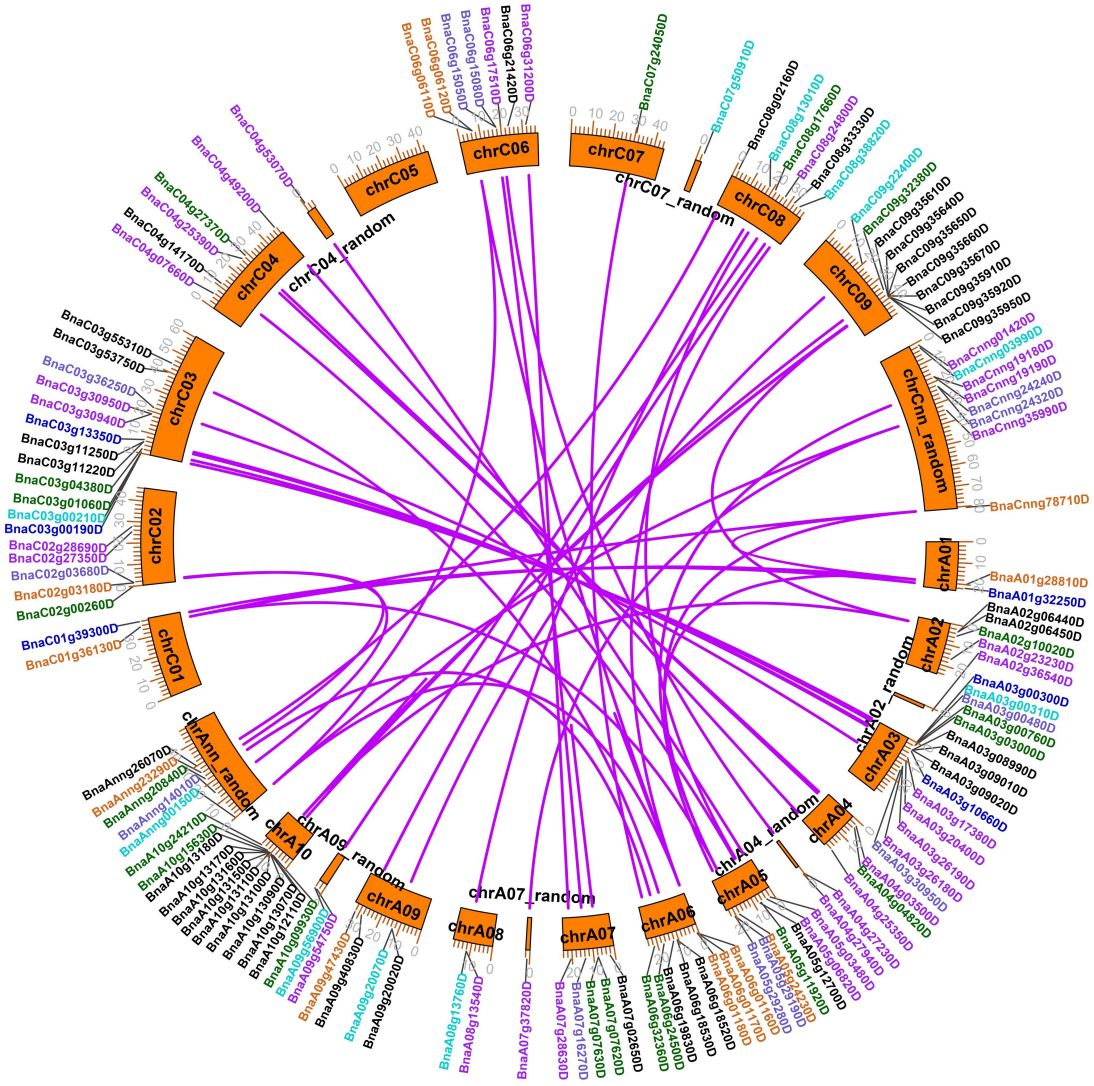
Chromosomal locations and duplicated gene analysis of *BnLegLu* genes in *Brassica napus*. The chromosomal locations of all *BnLegLu* genes are represented on different chromosomes, and different colors represent different *BnLegLu* subfamily genes. Subfamilies I to VII are indicated by black, purple, medium blue, dark turquoise, dark green, chocolate and slate blue color, respectively. Rose red lines are used to highlight the duplicated *BnLegLu* gene pairs.

A total of 130 legume lectin genes with an intact legume lectin domain in *B. napus* were identified using PF00139 as a query. The detailed information of each *BnLegLu* gene is presented in Supplementary Table S1. A total of 72 genes belonged to the A subgenome, whereas 58 genes belonged to the C subgenome (Supplementary Table S1). The protein length varied from 223 aa (amino acids; *BnaA06g01170D*) to 941 aa (*BnaC09g35640D*), with an average length of 528 aa, and the molecular weight (MW) varied from 24.96 to 103.59 kDa. The pI (isoelectric points) ranged from 4.68 to 9.99. Overall, the number of exons ranged from one to six, with an average of two exons, and 57 of the *BnLegLu* members only had one exon. Moreover, the instability index ranged from 13.76 to 51.48, and 72% (93 out of 130) *BnLegLu* members had an instability index lower than 40, indicating the stability of these proteins (Supplementary Table S1). Based on prediction with DeepTMHMM and CELLO server, 123 BnLegLu proteins contained SP (signal peptide) in their N-terminus; 107 members harbored the transmembrane domain (TM); and 103 members were localized in the plasma membrane (Supplementary Table S1), implying their possible functions in the plant-pathogen interaction on the cell surface. In addition, there are several genes containing multiple TM domains, such as *BnaC09g35640D*, *BnaC04g27370D* and *BnaA04g04820D*, which had three TM domains, and *BnaA03g26180D*, which had two TM domains (Supplementary Table S1). Moreover, the *BnLegLu* genes exhibited an uneven distribution across the chromosomes. More *BnLegLu* genes were distributed on chromosomes A03 (14 genes), C03 (12 genes), A10 (12 genes), C09 (10 genes), and A06 (eight genes), while fewer genes were found on chromosome A01 (two genes), C01 (two genes) and C07 (one gene; Supplementary Table S1; Figure 1). Duplication events of the *BnLegLu* genes were detected based on BLAST and MCScanX analyses of the genome of *B. napus* (Supplementary Table S1; Figure 1). There was a cluster of eight tandem repeats (*BnaA10g13070D* to *BnaA10g13180D*) on chromosome A10. A local duplication event was also identified on chromosome C09 (*BnaC09g35610D* to *BnaC09g35670D*; Figure 1). In total, 77.69% (101/130) of the *BnLegLu* genes originated from whole-genome duplication (WGD) or segmental duplication and 18 *BnLegLu* members were derived from dispersed duplication. Additionally, nine genes were derived from tandem duplication and two genes came from proximal gene duplication (Supplementary Table S1). Furthermore, 106 duplicated *BnLegLu* pairs between the two subgenomes were found in *B. napus* (Figure 1; Supplementary Table S2). Collectively, 130 *BnLegLu* genes in *B. napus* were identified and their detailed information, including gene characteristics and chromosomal distribution, was further elucidated.

### Phylogenetic analysis of *BnLegLus* in *Brassica napus*

**Figure 2:**
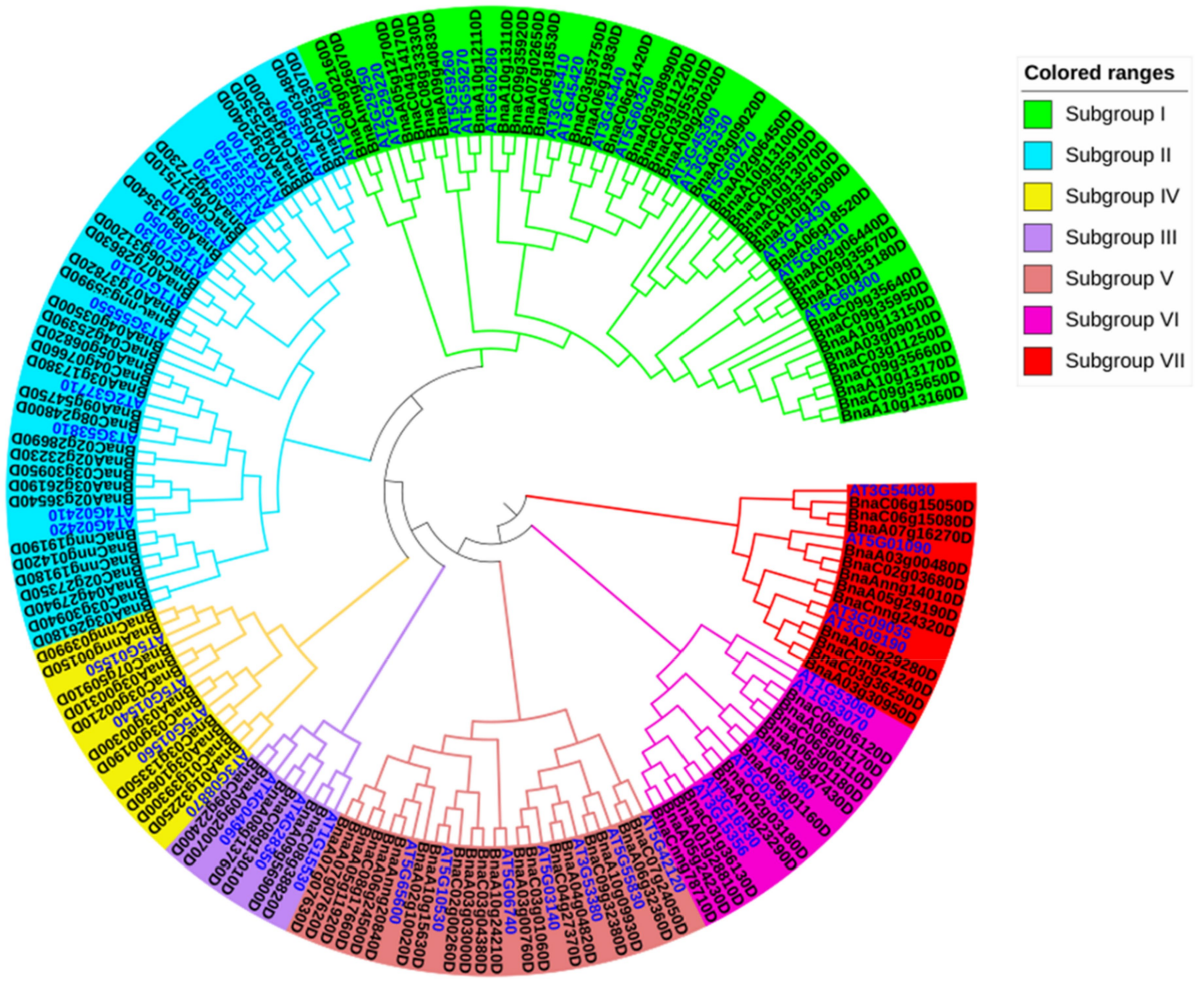
Phylogenetic analysis of BnLegLu proteins in *A. thaliana* and *B. napus*. All legume lectin proteins were clustered into seven subfamilies (I–VII) with differently colored branches (I, green; II, blue; III, purple; IV, yellow; V, dark red; VI, rose red; VII, red). The gene IDs for *B. napus* are black, the gene IDs for *A. thaliana* are bright blue.

To determine the phylogenetic classification of the *BnLegLu* genes, we constructed a phylogenetic tree of legume lectins by combing the LegLus proteins in *A. thaliana* (54; Eggermont et al., 2017) with the NJ (neighbor-joining) method (Figure 2). Seven (I–VII) clusters were categorized with well-supported bootstraps indication. The gene number varied significantly among the seven clusters, with the largest number of genes (54) in cluster I and the smallest number (9) in cluster III (Figure 2). Several well-studied *AtLegLu* genes were categorized into different clusters with *BnLegLu* genes, indicating that they possibly have distinct functions (Figure 2). Both *AtLecRK-I.9* (*AT5G60300*) and *AtLecRK-I.8* have been reported as immune elicitors in *A. thaliana* by binding NAD^+^, and inducing the expression of disease resistance genes (Wang et al., 2017). Nine genes (*BnaA1013160D*, *BnaC09g35650D*, *BnaA10g13170D*, *BnaC09g35660D*, *BnaC03g11250D*, *BnaA03g09010D*, *BnaA10g13150D*, *BnaC09g35950D* and *BnaC09g35640D*) and two genes (*BnaC09g35920D* and *BnaA10g13110D*) were located in the same clade with *AtLecRK-I.9* and *AtLecRK-I.8*, respectively (Figure 2). *AtLecRK-I.3* (*AT3G45410*) has been reported to be responsive to salt stress mediated by the ethylene signaling pathway (He et al., 2004). Phylogenetically, *BnaA06g18530D* and *BnaA07g02650D* were close to *AtLecRK-I.3* (Figure 2). *AtLecRK-V.5* (*AT3G59700*) and *AtLECRK-V.2* (*AT1G07130*) were found be involved in stomatal immunity (Arnaud et al., 2012; Desclos-Theveniau et al., 2012). In cluster II, *BnaC06g17510* and *BnaA04g27230* were grouped with *AtLecRK-V.5*, while *BnaC06g31200D* and *BnaA07g28630D* were grouped with *AtLECRK-V.2* (Figure 2). *BnaC09g22400D* and *BnaA09g20070D* in cluster III were closely related to *AtLecRK-VII.1* (*AT4G04960*), which controls stomatal immunity and jasmonate-mediated stomatal closure (Yekondi et al., 2018). The *BnLegLu* genes in cluster III were grouped with *LecRKA4.2* (*AT5G01550*), *LecRKA4.1* (*AT5G01540*), and *LecRKA4.3* (*AT5G01560*; Xin et al., 2009), indicating their possible functions in seed germination. Further, seven *BnLegLu* genes in cluster V (*BnaA07g07630D*, *BnaA07g07620D*, *BnaA05g11920D*, *BnaC08g17660D*, *BnaA06g24500D*, *BnaAnng20840D* and *BnaA02g10020D*) were clustered together with *AtLecRK-IX.2* (*AT5G65600*), which functions in plant PAMP-triggered immunity (PTI; Wang et al., 2015; Luo et al., 2017). In cluster VI, *BnaCnng78710D*, *BnaA05g24230D*, *BnaA01g28810D* and *BnaC01g36130D* were phylogenetically close to *AT3G16530* and *AtLEC* (*AT3G15356*), and the expression of *AT3G15356* is up-regulated by multiple stimuli such as developmental signal, wounding, jasmonate, ethylene, and chitin elicitor (Lyou et al., 2009). *BnaC02g03180D* and *BnaAnng23290D* were homologs of *AtSAI-LLP1* (*AT5G03350*), which is induced by salicylic acid (SA) and acts as a component of the SA-mediated defense processes associated with the effector-triggered immunity response (Armijo et al., 2013; Figure 2).

### Conserved structure of BnLegLus

To explore the evolutionary characteristics of *BnLegLus*, we further compared the gene structure and conserved motifs among the seven clades. The structures of most genes in cluster VI–VII were well conserved, and none of them contained any intron, except for *BnaA09g47430D* with one intron (Supplementary Table S1; Supplementary Figure S1). However, the genes in cluster I–V showed remarkable variations in intron number from zero to five as well as significant variations in intron length, with *BnaC09g35950D* and *BnaC03g11250D* having particularly long introns (Supplementary Table S1; Supplementary Figure S1).

Subsequently, we scanned the conserved motifs in all BnLegLu proteins and arranged them according to the gene phylogenic category (Supplementary Figure S2). The motifs 9, 6, 7 and 4, which correspond to the legume lectin domain, were highly conserved among all *BnLegLu* genes, while motifs1, 2, 3, 10 were annotated as kinase-related motifs and motifs 5 and 8 were unknown motifs (Supplementary Figure S2; Supplementary Table S3). Motif 7 was present in all the BnLegLu proteins except for BnaC06g3120D and BnaC04g25930D. Additionally, most genes contained motif 9 (124 out of 130) and motif 4 (120 out of 130; Supplementary Figure S2). The motifs in clusters VI and VII were well conserved without any kinase-related motif. All BnLegLu proteins in cluster VI contained four full motifs of legume lectin, while all BnLegLu proteins in cluster VII had no motif 6, and BnaAnng14010D, BnaC02g03680D and BnaA03g00480D only contained motif 9 and motif 7 (Supplementary Figure S2). All BnLegLu proteins in cluster III had full motifs of the protein kinase domain. These specific features implied the functional diversity of *BnLegLu* genes.

**Figure 3:**
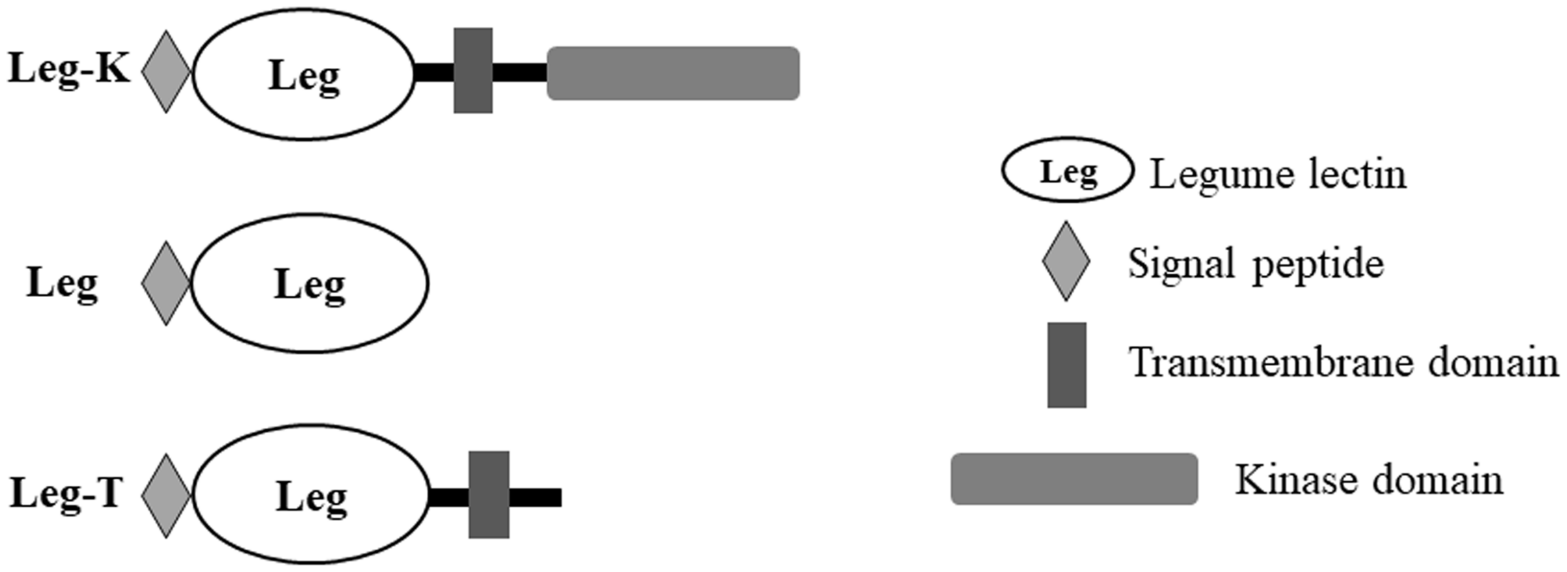
Schematic overview of the domain architecture in BnlegLus.

Moreover, an analysis of BnLegLu proteins with the domain search program of the Conserved Domain Database (NCBI)^1^ revealed that the BnLegLu proteins have relatively simple domain architecture, which could be divided into three categories: Leg-K representing BnlegLu proteins that contained SP, TM, legume domain and kinase domain, such as majority genes in cluster I–V; Leg representing BnlegLu proteins that only contained SP and legume domain, such as all genes in cluster VI except for BnaA06g01170D, which had no SP; Leg-T representing BnlegLu proteins that contained SP, TM and legume domain, like genes in cluster VII except for BnaCnng24320D and BnaA05g29190D, which had no TM (Figure 3; Supplementary Tables S1, S3). In addition, 92 out 130 of BnLegLus contained a kinase domain. All proteins that contained a protein kinase domain were synthesized with a signal peptide and a transmembrane domain, except for four genes (BnaA10g13090D, BnaC09g35610D, BnaA03g26180D and BnaA05g03480D) did not contain a signal peptide (Figure 3; Supplementary Tables S1, S3).

### *Cis*-element and protein interaction analysis of *BnLegLu* genes

To explore the possible regulatory mechanism of *BnLegLu* genes, we further analyzed the *cis*-elements in their promoter regions and the possible interactive proteins with the available public databases.^2^ The *cis*-elements in the 2-kb upstream region of *BnLegLus* were analyzed through the PlantCARE database. The results indicated the enrichment of stress- and hormone-related elements in these promoters, such as DRE core/DRE1 (involved in dehydration response), MBS (MYB binding site for drought stress), LTR (responsive to low temperature), TC-rich (defense responsive element), WUN-motif (responsive to wounds), ABRE (ABRE2/3a/4; responsive to abscisic acid), CGTCA/TGACG-motif (responsive to methyl jasmonic acid, JA), ERE (response to ethylene), p-box/TATC-box/GARE-motif (responsive to gibberellin), TGA-element/AuxRR-core (responsive to auxin), and TCA-element (responsive to salicylic acid; Supplementary Figure S3; Supplementary Table S4). Among the *cis*-elements, ABRE and JA-related elements were predominant in *BnLegLus*, as 97 and 101 out of the 130 *BnLegLu* genes contained ABRE and JA-related elements, respectively (Supplementary Figure S3; Supplementary Table S4). The category of *cis*-elements showed distinct patterns among different clusters. The elements of ABRE, JA-related, ERE and WUN-motif were abundant in the *BnLegLu* genes of cluster III and cluster VI, but the auxin-related, DRE-core, MBS and TC-rich elements were enriched in cluster III members. The *BnLegLu* genes in cluster I contained relatively more LTR elements, with a maximum of eight (*BnaC09g35640D*); while the *BnLegLu* genes in cluster V had more JA-related elements, with a maximum of 12 (*BnaA10g24210D*). The *BnLegLu* genes in cluster VII contained more GA-related and WUN-motif elements (Supplementary Figure S3; Supplementary Table S4). These results indicated the important functions of *BnLegLu* genes in response to environmental stress, which is possibly mediated by the hormone pathways.

**Figure 4:**
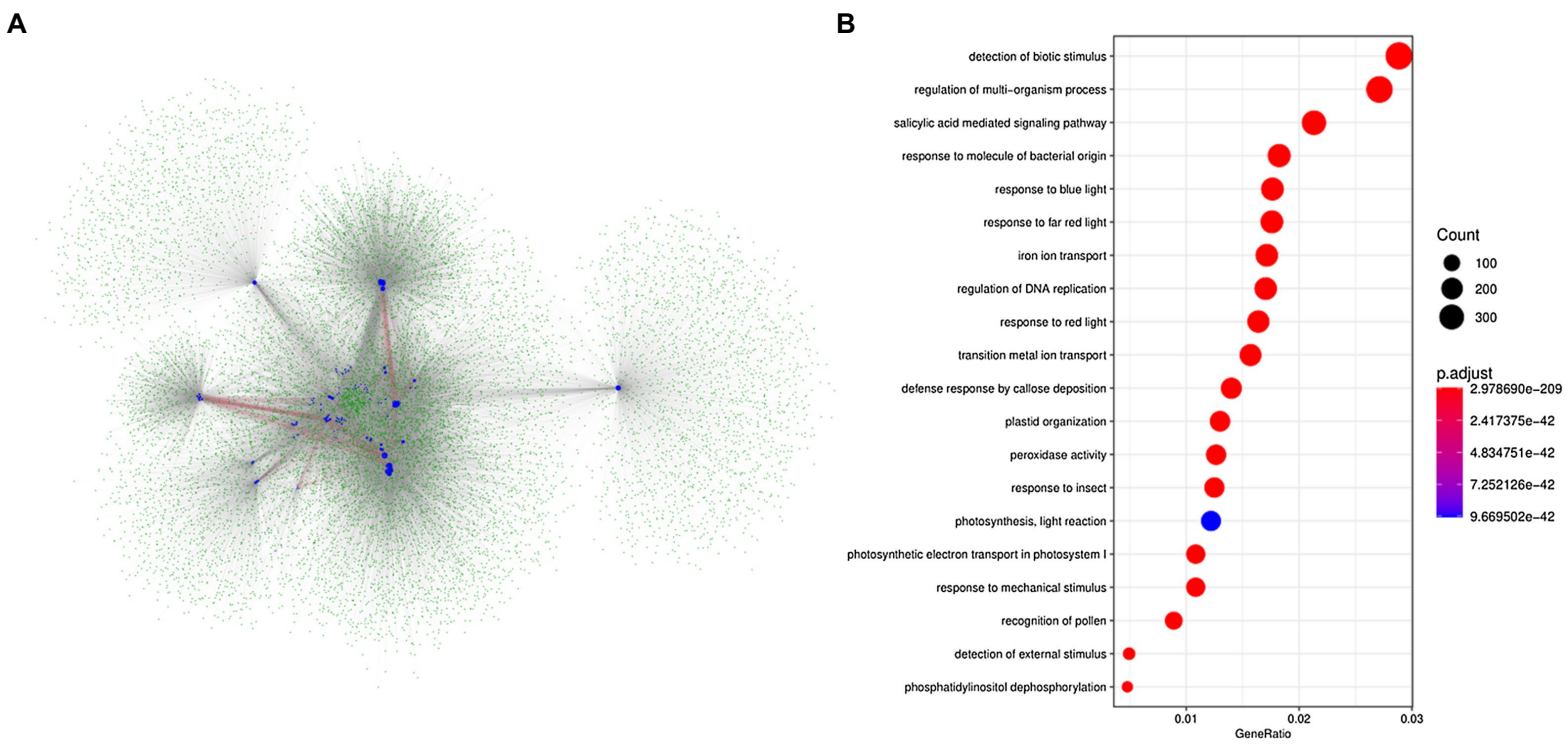
Analysis of the network for the interaction of BnLegLu proteins in *B. napus*. **(A)** Protein–protein interaction network of BnLegLu. The BnLegLu proteins are indicated by medium blue circles, and the green circles represent the proteins that interact with BnLegLu proteins. The red lines indicate the interaction between BnLegLu proteins, and the gray lines represent the interaction between BnLegLus and other proteins. **(B)** GO analysis of proteins interacting with BnLegLu proteins.

Based on the protein interaction networks in *A. thaliana*, we attempted to identify the potential interactive proteins of BnLegLus. By using the orthologs of BnLegLus in *A. thaliana* as the query, 4,606 putative interactive proteins were identified, which corresponded to 14,848 proteins in *B. napus*. Most BnLegLu proteins had interactions with each other (Figure 4A). To further elucidate the functional category of BnLegLu interactive proteins, we performed gene ontology (GO) and a Kyoto Encyclopedia of Genes and Genomes (KEGG) enrichment analysis. Interestingly, significant enrichment was found for the GO in terms of response to biotic and mechanical stimuli, insects, molecules of bacterial origin and the defense response by callose deposition, suggesting their important roles in plant defense (Figure 4B; Supplementary Table S5). In addition, salicylic acid-mediated signaling pathways, regulation of DNA replication and multi-organism process were also remarkably enriched, implying their important roles in plant development and signal transduction (Figure 4B; Supplementary Table S5). The KEGG enrichment analysis revealed that these interactive proteins participate in photosynthesis, glycolysis/gluconeogenesis and fundamental metabolisms such as glutathione metabolism, inositol phosphate metabolism and glyoxylate and dicarboxylate metabolism (Supplementary Figure S5).

### Expression analysis of *BnLegLus* in various tissues during different developmental stages

**Figure 5:**
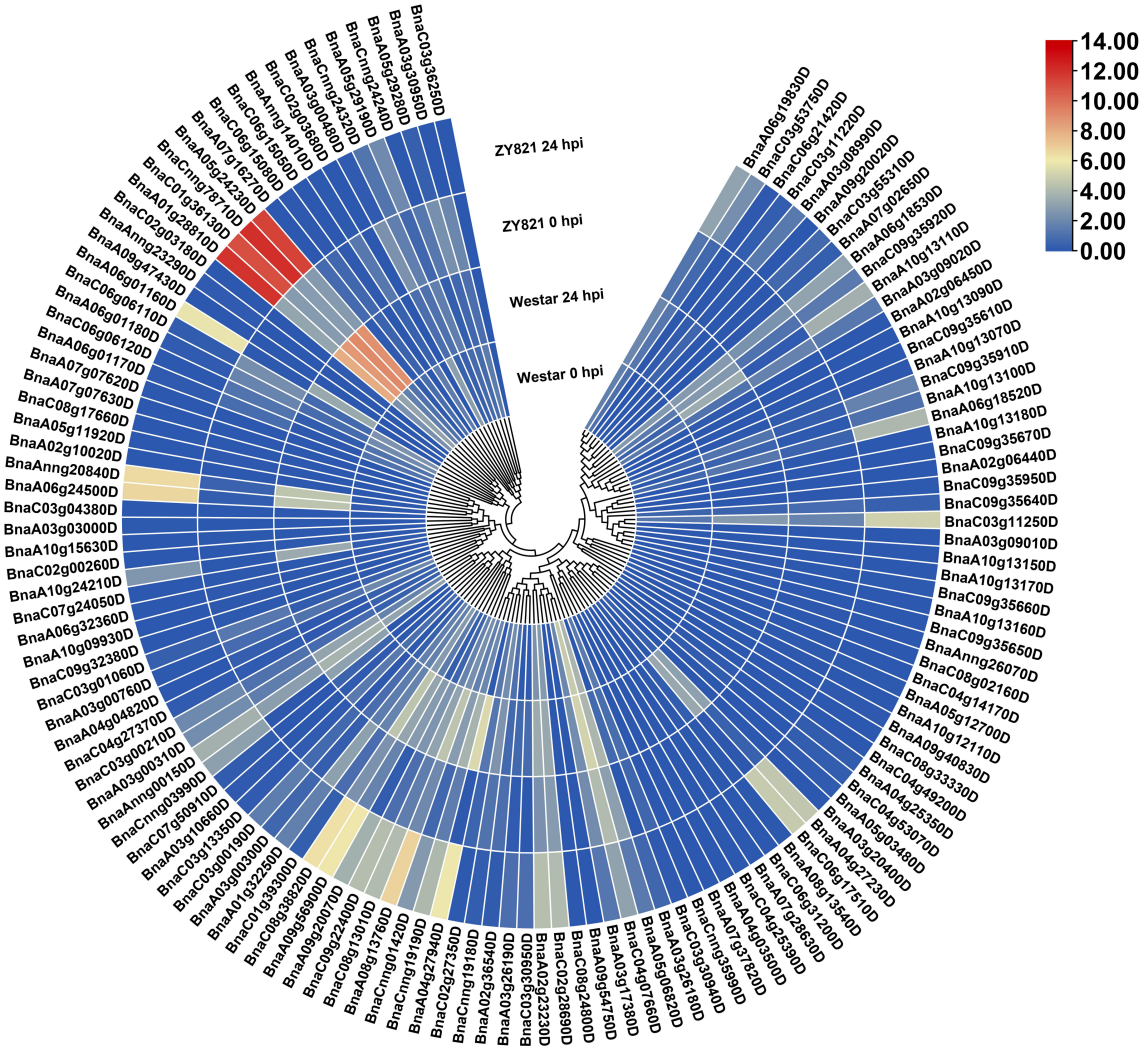
Expression patterns of the *BnLegLu* genes in Westar and ZY821 cultivars at 0 and 24 h after *Sclerotinia sclerotiorum* inoculation. The heatmap was generated by taking log2 fold of FPKM values. The color bar shows the relative expression from low (blue) to high (red).

To investigate the expression patterns of *BnLegLus*, comprehensive expression analysis was performed by using the published transcriptome information resource (Liu et al., 2021b).^3^ This database collected 92 tissue samples at three distinct developmental stages (leaf, silique wall and seed), including the cotyledon, root, stem peel, leaf, bud, flower, silique, silique wall and seed. The FPKM (fragments per kilobase million) values for each *BnLegLu* gene are presented in Supplementary Table S6 and the expression profiles are clustered in Supplementary Figure S5. In general, 25 out of the 130 genes were barely expressed across tissues (FPKM < 0.5). Moreover, most *BnLegLu* genes showed moderate expression (FPKM < 15), and only several genes were highly expressed in some specific tissues (Supplementary Figure S5; Supplementary Table S6). A comparison of the expression levels among different tissues revealed that *BnLegLus* had high expression in roots, old leaves and mature silique walls, but were barely expressed in the pollen. For example, four genes of *BnaA05g24230D*, *BnaC04g07660D*, *BnaCnng24320D* and *BnaC06g06120D* showed the highest expression in the root, old leaf, silique wall and seed (Figure 5; Supplementary Table S6). In addition, the expression of *BnLegLu* genes in cluster I–V in old leaves and silique walls continuously increased along with development, except for some unexpressed or lowly expressed genes (Supplementary Figure S5; Supplementary Table S6), while those genes in cluster VI–VII exhibited high expression in the early stage of seed development. In summary, the expression of *BnLegLus* varied greatly among different tissues, implying their functional diversity in the development of *B. napus*.

### Expression analysis of *BnLegLus* under abiotic stress and *Sclerotinia sclerotiorum* induction

To gain insights into the response of *BnLegLus* to environmental stimuli, we examined the expression fluctuations of *BnLegLus* under various abiotic stresses (dehydration, cold, ABA and salinity treatments; Zhang et al., 2019). The majority of *BnLegLu* genes had very low levels of expression in the control, which was inconsistent with tissue expression data. Abiotic stress significantly changed the expression levels of 35 *BnLegLu* genes (≥2-fold and FPKM ≥ 2), among which 30 were up-regulated and five were down-regulated (Supplementary Figure S6; Supplementary Table S7). The *BnLegLu* genes in cluster I and cluster IV, which showed low expression in different tissues, exhibited minor fluctuations in the transcript after induction by abiotic stress (Supplementary Figure S6; Supplementary Table S7). However, six *BnLegLu* genes in cluster II (*BnaC04g07660D*), VI (*BnaA01g28810D*, *BnaC01g36130D*, *BnaCnng78710D* and *BnaA05g24230D*) and VII (*BnaCnng24320D*) showed a significant response to all stress conditions (Supplementary Figure S6; Supplementary Table S7). Compared with that in the control, the expression of *BnaC04g07660D* and *BnaCnng24320D* was increased to 6.4-fold and 53-fold in 24 h of cold treatment; that of *BnaA01g28810D* and *BnaC01g36130D* was elevated to 70-fold and 24-fold under salt stress in 24 h; that of *BnaCnng78710D* and *BnaA05g24230D* was increased to 3.1-fold and 3.7-fold in 4 h of cold treatment, but their expression was significantly down-regulated after 24 h of ABA treatment and NaCl treatment (Supplementary Figure S6; Supplementary Table S7). Furthermore, we also analyzed the expression changes of *BnLegLus* under the induction of *S. sclerotiorum* by utilizing the public transcriptome data (Girard et al., 2017). More than half of the *BnLegLu* genes showed very low levels of expression (FPKM < 0.5) in the two cultivars of Zhouyou821 (resistant to SD) and Westar (susceptible to SD), except for *BnaC04g07660D*, which had a FPKM value higher than 15 (Figure 5; Supplementary Table S8). A total of 40 *BnLegLu* genes showed significant changes in expression under the induction of *S. sclerotiorum* (≥2-fold and FPKM ≥ 2), including 30 up-regulated genes and 10 down-regulated genes at 24 h post-inoculation (hpi) at least in one cultivar (Figure 5; Supplementary Table S8). Compared with the down-regulation, the up-regulation seemed to be more significant, moreover, the expression levels of most *BnLegLu* genes changed more significantly in ZY821 than those of Westar (Figure 5; Supplementary Table S8). Relative to those in cluster I, II, IV, V and VII, the genes in cluster III and VI showed more remarkable up-regulation of expression in response to *S. sclerotiorum*. Six genes (*BnaA08g13760D*, *BnaC08g13010D*, *BnaC09g22400D*, *BnaA09g20070D*, *BnaA09g56900D* and *BnaC08g38820D*) in cluster III were induced by *S. sclerotiorum* and more significantly induced that of four genes (*BnaA01g28810D*, *BnaC01g36130D*, *BnaCnng78710D* and *BnaA05g24230D*) in cluster VI, with the most significant up-regulation reaching 715-fold (Figure 5; Supplementary Table S8).

**Figure 6:**
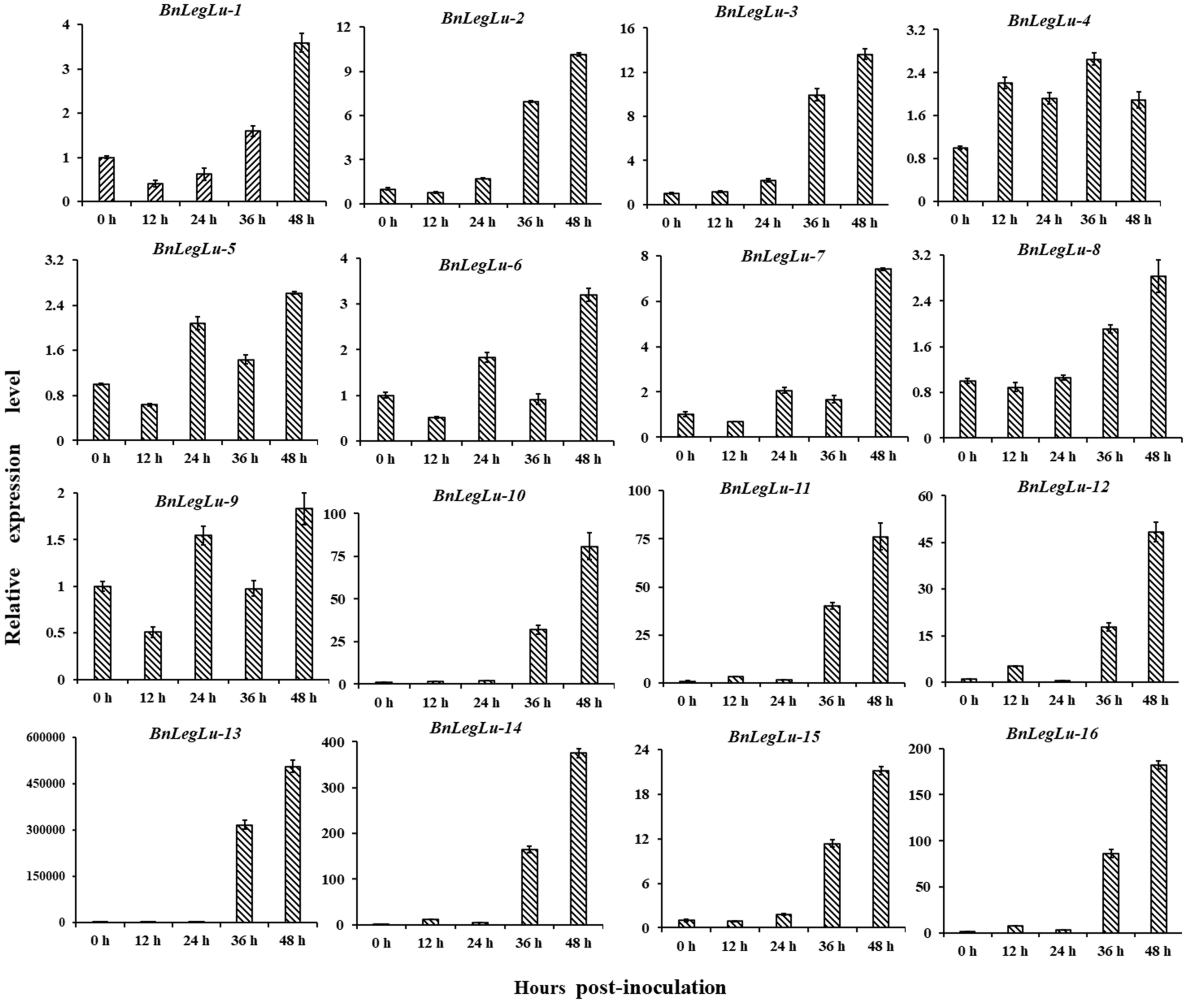
Expression validation of 16 candidate *BnLegLu* genes in response to *S. sclerotiorum* determined by qRT-PCR. The time points 0, 12, 24, 36 and 48 h represent hours after inoculation with *S. sclerotiorum*. The error bars show the standard error of three replicates.

Next, we selected 16 candidate *BnLegLu* genes with particular increases in expression after *S. sclerotiorum* induction for real-time PCR (qRT-PCR) analysis, including *BnaA04g27940D*, *BnaA04g27230D*, *BnaC06g17510D*, *BnaA09g56900D*, *BnaC08g38820D*, *BnaA08g13760D*, *BnaC08g13010D*, *BnaC09g22400D*, *BnaA09g20070D*, *BnaA06g24500D*, *BnaAnng20840D*, *BnaA06g01160D*, *BnaA01g28810D*, *BnaC01g36130D*, *BnaCnng78710D* and *BnaA05g24230D*, which were renamed as *BnLegLu-1* to *BnLegLu-16*. We collected the leaves of Zhouyou821 after inoculation with *S. sclerotiorum* at different time points (0, 12, 24, 36 and 48 h; Figure 6). As a result, four genes (*BnLegLu-4*, *BnLegLu-5*, *BnLegLu-6* and *BnLegLu-9*) showed complex changes in expression. After inoculation, the expression of *BnLegLu-4* was generally up-regulated all the time with a slight decrease at 24 and 48 hpi, while that of the other three genes was down-regulated at 12 hpi first, and then continuously increased, but slightly decreased at 36 hpi (Figure 6). The remaining 12 genes were continuously up-regulated at all times after infection, which was consistent with the transcriptome data. The expression of *BnLegLu-10* to *BnLegLu-16* was very remarkably up-regulated, even by up to 50,000-fold (*BnLegLu-13*; Figure 6). These results indicated that these genes may play potential roles in the SD resistance of *B. napus*.

### Preliminary validation of candidate *BnLegLus* for SD resistance in *Brassica napus*

**Figure 7:**
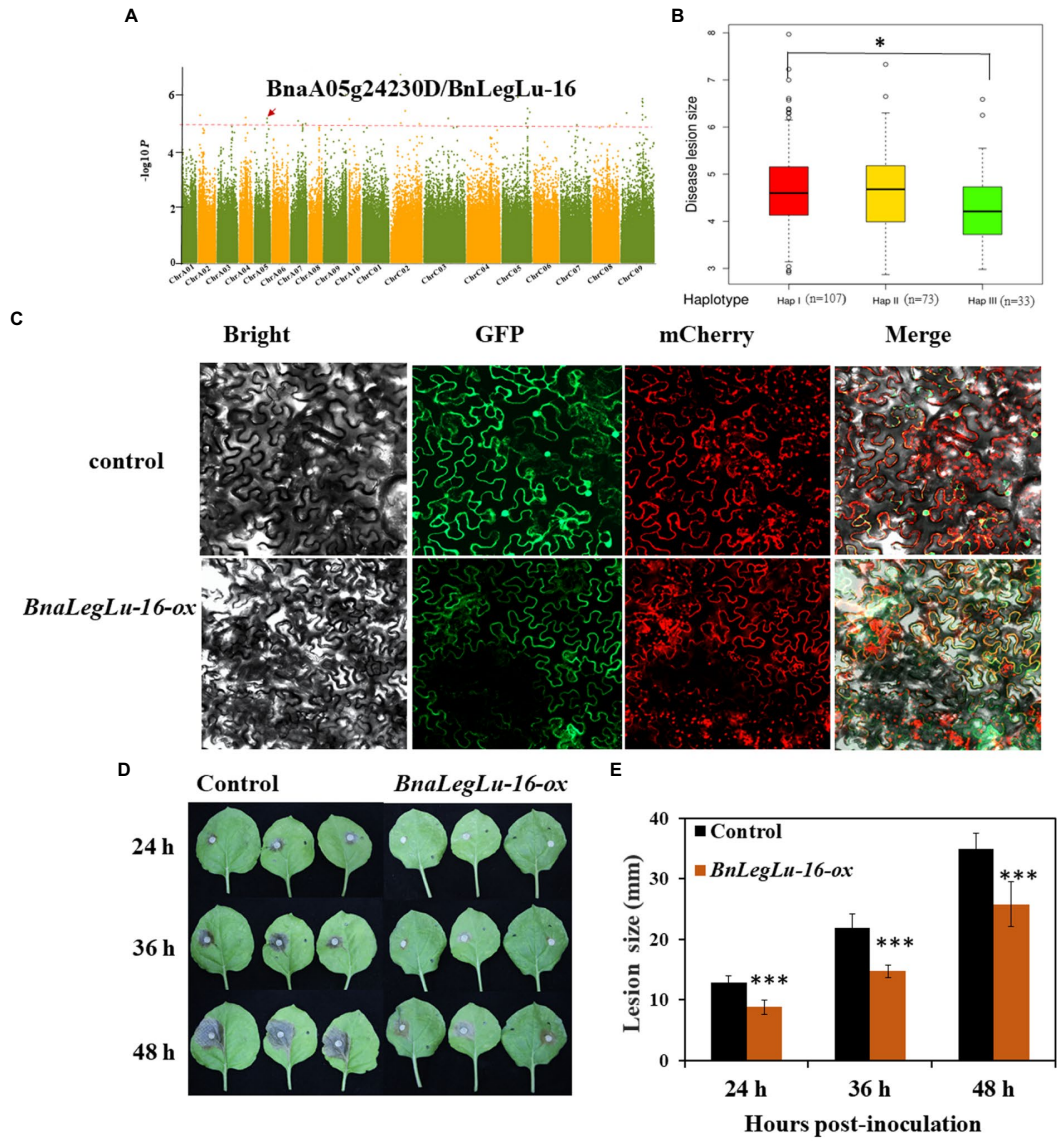
Functional validation of *BnLegLu-16* for SD resistance. **(A)** Genome-wide association analysis (GWAS) for SD resistance in a *B. napus* population comprising 324 accessions and Manhattan plots of SD resistance from GWAS; Manhattan plots of the disease after 36 h; the red dashed line shows GWAS threshold (1/SNP number). **(B)** Haplotype analysis of *BnLegLu-16*. **(C)** Subcellular localization of BnLegLu-16-ox in tobacco, BnLegLu-16-GFP fusion protein was transiently co-expressed with the mCherry–H^+^-ATPase, which was used as the plasma membrane marker in tobacco leaves. GFP and mCherry were transiently co-expressed as the control. After 48 h post-incubation, the tobacco leaves were observed with confocal microscope (ZEISS LSM710, Germany). **(D)** Disease lesion sizes on leaves at 24, 36 and 48 hpi with *S. sclerotiorum*, which was transiently injected through agrobacterium containing *BnLegLu-16-ox* and the control into tobacco leaves. **(E)** Disease lesion sizes statistically analyzed by comparing the overexpression *BnLegLu-16-ox* to the control. The data represent the means ± 2 SD from three independent experiments, with each containing 20 leaves. Significant differences in lesion size between *BnLegLu-16-ox* and the control are indicated (Student’s *t*-test) as follows: ***p < 0.001). The error bars show the standard error of three replicates.

Based on the available GWAS data in our laboratory for SD disease in the panel of 324 accessions collected worldwide (Tang, 2019; Ding et al., 2020), we detected four *BnLegLu* genes located in the region of significant SNPs associated with SD, including *BnLegLu-1* (81 kb near the associated SNP of 391,255 bp on chromosome A04, *BnLegLu-2* (88 kb near the associated SNP of 92,114 bp on chromosome A04), *BnLegLu-5* (14 kb near the associated SNP of 34,896,206 bp on chromosome C08), *BnLegLu-16* (77 kb near the associated SNP of 18,259,474 bp on chromosome A05; Figure 7A; Supplementary Figure S7). We also analyzed the haplotype of *BnLegLu-16* and three haplotypes divided by the SNPs in *BnLegLu-16* is displayed in Figure 7B and Supplementary Table S10. The result showed that the disease lesion size in haplotype I and III exhibited significant difference (*p* = 0.02). Furthermore, we transiently overexpressed *BnLegLu-16* in tobacco leaves, and inoculated them with the *S. sclerotiorum* hyphal plugs on leaves. Compared to the control, the disease lesion size was significantly small on leaves with overexpression of *BnLegLu-16*, and the difference was increasing obviously along with the time post-inoculation (Figures 7D,E). We further examined the subcellular localization by detecting the fluorescence under confocal microscopy. Results showed that the GFP-tagged BnLegLu-16 (BnLegLu-16–GFP) displayed an obvious signal in the plasma membrane, while the GFP-only control produced a fluorescence signal throughout the cell (Figure 7C). Furthermore, BnLegLu-16-GFP co-localized with the mCherry-tagged H^+^-ATPase (Sun et al., 2021), which was used as a marker for plasma membrane location (Figure 7C). The results showed that the fluorescence was specifically present on the cell membrane (Figure 7C), indicating the possible function compartment of *BnLegLu-16*.

## Discussion

Legume lectins were extensively distributed in the plant, and display a variety of functions including antimicrobial and insecticidal activities (Lagarda-Diaz et al., 2017; Katoch and Tripathi, 2021). Increasing evidence has suggested that insect and pathogen infestation of crops is one of the crucial factors threatening global agriculture production, whose impacts will further increase in the future due to increasing demand for food and changing climatic conditions (Fedoroff et al., 2010). Besides, crops will be confronted with stress more often and different stresses will occur in combination more frequently (Saijo and Loo, 2020). Hence, as potential candidates for resistance to susceptible crops, legume lectins are worth in-depth and extensive investigation. In the last two decades, much research attention has been paid to legume lectins that are weakly expressed in non-storage tissues (Katoch and Tripathi, 2021; Van Damme, 2022). It has been found that *AtLecRK-V.5*, *AtLecRK-IX.2* and *AtLecRK-I.9* contribute to plant resistance to *P. syringae* (Desclos-Theveniau et al., 2012; Balagué et al., 2017), and *AtLecRK-VI.2* and *AtLecRK-I.9* play important roles in plant immune response (Huang et al., 2014). The interaction between *S. sclerotiorum* and its host *B. napus* is very complex due to the facultative parasitic nature of *S. sclerotiorum* (Ding et al., 2021). In addition, no immune germplasm has been identified in *B. napus* or its close relatives so far, bringing about great challenges to the breeding of *B. napus* for SD resistance (Wu et al., 2016). Numerous studies have shown that there are many potential genes with the ability to resist SD in *B. napus*. Moreover, the heritability of the resistance of *B. napus* to *S. sclerotiorum* is moderate (60–75%; Ding et al., 2021). These resistance genes can be mainly divided into three categories: (i) Some genes can produce phytoalexins (secondary metabolites) to kill the pathogen or prevent pathogen infection and spread during induction of *S. sclerotiorum*, such as flavonoids, terpenoids, indole alkaloid camalexin, glucosinolates, lignin (Cao et al., 2022), and brassinosteroids (BR; Liang and Rollins, 2018). (ii) Some genes can generate activating enzymes or antimicrobial peptides to degrade the cell wall of *S. sclerotiorum* or block or interfere with its pathogenicity factors, such as *BnPGIPs*, which could inhibit hyphal extension and plant cell wall degradation through PG secreted by *S. sclerotiorum* (Wang et al., 2018a). (iii) Some genes can activate the defense signaling pathways involving the MAPK (mitogen-activated protein kinase) cascade reaction, ROS (reactive oxygen species), SA (salicylic acid), and JA (jasmonic acid) to activate the resistance to *S. sclerotiorum* (Nováková et al., 2014), such as *BnWRKY33*, *BnWRKY70*, *BnMPK3*, *BnMPK4* and *BnMPK6* (Liu et al., 2018). In general, great progress has been made in improving SD resistance in rapeseed, but the key genes for the resistance remain to be explored. The *LegLu* genes are an important disease resistant gene family with antimicrobial and insecticidal activities, but their role in SD resistance remains largely unknown. In this study, we performed a comprehensive analysis of *BnLegLus* at the genome level and analyzed their evolutionary characteristics and functional impact on SD resistance. The findings provide new insights into this gene family and help to predict its potential function in plant stress response, particularly in SD resistance.

In this study, we identified 130 *BnLegLu* genes in *B. napus* by using the *Darmor-bzh* v4.1 genome sequence information (Supplementary Table S1). According to the phylogenetic analysis, the *BnLegLu* genes could be classified into seven clusters together with *AtLegLus* (Figure 2). The majority of *BnLegLu* genes in cluster I–V contained extended RLKs domains, which could explain the higher molecular weight of *BnLegLu* genes in these clusters than those in cluster VI–VII. Although *BnLegLu* genes in both cluster VI and VII contained the legume lectin domain, the members in cluster VII generally had higher average molecular weights than those in cluster VI due to the additional transmembrane domain. In terms of size polymorphism, the *BnLegLu* genes also showed variations in exon number, pI, instability index and subcellular localization in three categories (Figure 3; Supplementary Table S1). All *BnLegLu* genes in Leg and Leg-T only contained one exon except for *BnaA09g47430D*, while the number of exons ranged from one to six in Leg-K (Supplementary Table S1; Supplementary Figure S1). The average pI of BnLegLu proteins in Leg-K, Leg and Leg-T was 6.88, 8.08 and 6.89, and the instability index was 42.4, 21.4 and 37.98, respectively. Prediction of subcellular localization revealed that the *BnLegLu* genes in Leg-K, Leg and Leg-T were mainly located in the plasma membrane, extracellular, and chloroplast/plasma membrane, respectively (Figure 3; Supplementary Table S1). The observed variations in the characteristics of *BnLegLus* are probably the result of a series of evolutionary processes. Tandem and segmental duplications have been identified as the major mechanisms driving the expansion of lectins in soybean and rice (Jiang et al., 2010; Van Holle and Van Damme, 2015), which was also observed in *B. napus*. Most of *BnLegLu* genes (101 of 130) originated from WGD or segmental duplication, implying that this gene family is associated with the adaptation to various environmental stresses through duplication and divergence (Figure 1; Supplementary Table S1). In general, the fusion of protein and extended kinase domains represents the development of plant adaptation to stress (Bailey et al., 2018). All identified types of LecRLK can be considered as plant defense-related proteins as they might act as a receptor at the level of the cell wall/plasma membrane of the plant cell during a pathogen attack (Bellande et al., 2017). However, the functionality of these lectins needs to be investigated in more detail in the future.

Given that some lowly expressed lectins are not constitutively expressed but induced by environmental stresses, we investigated the spatiotemporal expression patterns of *BnLegLu* genes. Although the expression level of most *BnLegLu* genes was low, it showed great differentiation during the growth and development of *B. napus* (Supplementary Figure S5). A considerable number of genes play important roles in leaf development, silique wall development and seed development, such as some *BnLegLu* genes in cluster II, III and IV, whose expression level continuously increased at the leaf and silique wall developmental stage; some *BnLegLu* genes in cluster VI and VII had high expression in the early stage of seed development (Supplementary Figure S5). An *in silico* analysis of *BnLegLus* under abiotic and SD induction revealed that nearly 30% of *BnLegLu* genes are responsive to at least one stress (Figure 5; Supplementary Figure S6). Among these genes, those in cluster III and VI exhibited relatively high expression after induction by at least one stress (Figure 5; Supplementary Figure S6). Moreover, 16 *BnLegLu* genes were confirmed to be responsive to *S. sclerotiorum* as indicated by real-time PCR (qRT-PCR) analysis (Figure 6). In addition, *cis*-element promoter analysis also demonstrated that the *BnLegLu* genes in cluster III and VI contained more hormone-related and plant defense-related elements such as auxin-related, DRE-core, MBS and TC-rich elements (Supplementary Figure S3). Furthermore, the GO and KEGG pathway analysis predicted that the BnLegLu proteins are involved in response to biotic, mechanical stimuli and insects (Figure 4; Supplementary Figure S4). Overall, the expression of *BnLegLus* varied greatly among various stages of plant development, implying their essential roles in the development of *B. napus*, particularly in SD resistance, which may be an important adaptation strategy to the environment.

The mechanism for the response of *BnLegLus* to stress may be different in Leg-K, Leg and Leg-T. According to the phylogenetic analysis with the already known functions of *AtLegLu*, the *BnLegLu* genes in Leg-K may play important roles in plant immune response (Yekondi et al., 2018), while the *BnLegLu* genes in Leg may function in SA-mediated defense processes associated with the effector-triggered immunity response (Armijo et al., 2013; Figure 2). The role of *BnLegLus* in Leg-T remains unclear, as the function of their homologs in *A. thaliana* is unknown (Figure 2). The *BnLegLu* genes containing the kinase domain in cluster III may respond to *S. sclerotiorum* through stress signal transduction (Bellande et al., 2017); while the four *BnLegLus* genes in cluster VI whose expression was induced by SD may recognize and respond to *S. sclerotiorum* hyphae as a function of classical lectins (De Coninck and Van Damme, 2021; Fonseca et al., 2022; Figure 2). Previous studies have shown that lectins can bind to hyphae, resulting in the swelling of hyphae, vacuolation of the cell content, destruction of the nutrient absorption of the fungi, interference with spore germination, and even cell wall lysis (Lagarda-Diaz et al., 2017; Katoch and Tripathi, 2021). Here, subcellular localization analysis revealed that *BnLegLus-16* was localized on the cell membrane, and its overexpression enhanced SD resistance in tobacco (Figures 7C–E), which provides a causal gene contributing to SD resistance in the plant. To evaluate the function of *BnLegLus-16* on SD resistance in *B. napus*, the stable transformants with overexpression of *BnLegLus-16* in *B. napus* and its close relative of *A. thaliana* is needed. In addition, the elaborate work of *BnLegLus-16* on cell membrane during *S. sclerotiorum*-*B. napus* interaction also needs to be further emphasized.

## Materials and methods

### Identification of *BnLegLu* gene family in *Brassica napus*

In order to identify the *BnLegLu*, we used PF00139 as a query from Pfam database^4^ (Mistry et al., 2021) to search for *LegLu* genes in all proteins of *B. napus* (the E-value was set to 1e-5) by HMMER3.0.^5^ Then, the SMART database^6^ and Conserved Domain Database (NCBI; Lu et al., 2020)^7^ were used for verification of the candidate genes. The redundant *BnLegLu* genes were manually excluded. The current genome sequence and annotation information of rapeseed cultivar “Darmor-bzh” was obtained from the Brassicaceae Database (Chalhoub et al., 2014).^8^ The legume lectin genes in *A. thaliana* were downloaded from the Tair database.^9^

Peptide length, molecular weight, isoelectric point and instability index of each BnLegLu protein were calculated with the online ExPasy program.^10^ Signal peptide and transmembrane domain were predicted by online DeepTMHMM program (Singh et al., 2011).^11^ Subcellular localization was predicted by online CELLO v.2.5 program (Yu et al., 2004).^12^ The physical locations of *BnLegLus* on the chromosomes were obtained from the annotation of the *B. napus* genome. To identify the gene duplication events, BLASTP was used with the E-value of 1 × 10^−10^ to align the sequence and MCScanX was used to detect the duplication patterns, including segmental and tandem duplication. The chromosomal locations and duplication events were visualized using the TBtools software (Chen et al., 2020).

### Phylogenetic analysis of *BnLegLu* gene family

The legume lectin protein sequences of *A. thaliana* and *B. napus* were subjected to multiple sequence alignment using the ClustalW2 (Larkin et al., 2007) to acquire insights into the evolutionary relationships between *BnLegLu* members. Phylogenetic trees were generated with the MEGA7 (Kumar et al., 2016) program using the Neighbor-Joining (NJ) method with 1,000 bootstrap replications. The tree was visualized using iTOL v6.5.2.^13^

### Analysis of gene structures and protein conserved domains of *BnLegLu* gene family

The information of gene structures for *BnLegLus* was obtained from GFF files. Multiple Expectation Maximization for MEME (Motif Elicitation 5.4.1; Bailey et al., 2009) was used to analyze the conserved motifs in BnLegLu proteins. To this end, the following parameters were calibrated: maximum 10 motifs, with an optimal width of 6–50 amino acids. The remaining parameters were set to their default values. The identified motifs were annotated by using the Pfam database.^14^ The Conserved Domain Database (NCBI)^15^ was used to analyze the conserved domain of BnLegLu proteins. The TBtools was used to visualize the gene and motif structures (Chen et al., 2020).

### *Cis*-element and protein interaction analysis of *BnLegLus*

The promoters of *BnLegLus* (2-kb upstream sequences from the initial codon) were extracted to identify the cis-acting regulatory elements using PlantCARE.^16^ The protein–protein interaction (PPI) of BnLegLu proteins in *A. thaliana* was downloaded from STRING (Szklarczyk et al., 2019).^17^ The functional association networks of the BnLegLu proteins in *B. napus* were predicted based on the homologs in *A. thaliana*, and Cytoscape (Shannon et al., 2003) was used to display the interactions. The genes interacting with the BnLegLu proteins were taken for gene ontology and KEGG enrichment analysis using the cluster Profiler in R (Yu et al., 2012).

### Expression analysis of *BnLegLus* under different conditions

The transcriptome data from 92 tissue samples (eight different tissues covering ZS11 and three distinct developmental stages, including cotyledon, root, stem peel, leaf, bud, flower, silique, silique wall and seed; Liu et al., 2021b), four treatments (dehydration, cold, ABA and salinity; Zhang et al., 2019) and two cultivars (susceptible *B. napus* cv. Westar and tolerant *B. napus* cv. Zhongyou 821) induced by *S. Sclerotiorum* (Girard et al., 2017) were used in this study. The expression levels of *BnLegLu* genes were calculated with stringtie. Finally, the FPKM values were converted to log2 fold, and heat maps of all data were displayed by TBtools (Chen et al., 2020).

The seeds of ZY821 (from our laboratory) were germinated and grown in a growth room at 22°C with a 16-h light and 8-h dark photoperiod. *S. Sclerotiorum* strains (separated from the stubbles of oilseed rape in the fields of Yangluo, Wuhan, designated as WH-13) were used in this study. First, the fungal strains preserved at 4°C were sub-cultured on potato dextrose agar medium. Then, the new marginal hyphae were excised using a 7-mm puncher and closely upended onto the adaxial surface of healthy leaves (five-leaf stage). The inoculated plants were placed in a humidification chamber to keep the humidity above 85%. Each plant was inoculated with three leaves, and samples were taken every 12 h and immediately stored in liquid nitrogen. For each biological replicate, lesions were pooled from a minimum of three different plants and ground to a powder in liquid nitrogen. Total RNA was extracted by using Invitrogen TRIZOL Reagent.^18^ First-strand complementary DNA (cDNA) was synthesized by TaKaRa reverse transcription kit.^19^ Quantitative RT-PCR (qPCR) was performed using Bio Supermix^20^ following the manufacturer’s instructions. Reaction steps were performed with the following program: 95°C for 3 min; 40 cycles of 95°C for 15 s; 56°C for 15 s followed by 65°C for 5 s and 95°C for 5 s. The *B. napus* β-actin gene (AF111812) was used as a reference standard. All experiments were performed in three biological replicates. The relative expression was calculated using the 2 − ∆∆Ct method (Livak and Schmittgen, 2001).

### Functional analysis of *BnLegLus* by genome-wide association analysis and transgenic strategy

To screen the potential *BnLegLu* genes responsible for SD resistance, the candidate genes based on the GWAS results obtained previously in our laboratory were checked (Tang, 2019; Ding et al., 2020). The GWAS population included 324 rapeseed accessions with different resistance levels collected worldwide. Resequencing of the GWAS accessions was performed by the commercial Illumina HiSeqXTen service (BGI-Shenzhen, China). For SD resistance determination, the leaves of plants grown in a field in Wuhan in 2015 at the stage of three-to-four leaves were excised and incubated in a growth room after inoculation with *S. Sclerotiorum*. The disease lesion size was examined at 12, 24, 36 and 48 hpi.

Open reading frame of *BnLegLus*-16 was cloned from ZY821 into pCambia2300-GFP at Bam-HI and KpnI sites using the ClonExpress II One Step Cloning Kit (Vazyme Biotech Co., LTD, Nanjing, China; primers are shown in Supplementary Table S9). Agrobacterium (GV3101 competent cell) containing corresponding plasmids was infiltrated into four-week-old tobacco (*Nicotiana. L*) leaves for transient expression of fusion protein. Every tobacco was injected with three leaves and each plasmid was injected with 15 plants. These plants were incubated at 22°C in a growth room with a 16-h light and 8-h dark photoperiod for 60 h (Sparkes et al., 2006). For an accurate subcellular localization, the mCherry–H^+^-ATPase fusion protein, a marker of plasma membrane protein (Sun et al., 2021), was co-expressed with the BnLegLu-16–GFP, the fluorescence of tobacco leaves was monitored by confocal microscopy (ZEISS LSM710, Germany). The fluorescence emissions were at 510–540 nm for eGFP or at 600–650 nm for mCherry and excitations were at 450–490 nm for eGFP or at 523–588 nm for mCherry. Then, the leaves (expressed *BnLegLus*-16-ox and empty GFP vector, respectively) were excised and inoculated with *S. Sclerotiorum* hyphal plugs on detached leaves in the chamber with the humidity >85% and cultured in darkness. Each leaf was inoculated with one mycelium block. The size of disease spots was measured and photographed every 12 h. Then, the data were statistically analyzed, and three replicates were set for each experiment.

## Conclusion

In this study, we conducted detailed characterization and investigation of *BnLegLu* genes. In total, 130 *BnLegLu* genes were identified and phylogenetically categorized into seven clusters. The genes in cluster I–V were variable in terms of gene number, structure and conserved motif, while those in cluster VI–VII were highly conserved. The majority of *BnLegLu* genes in cluster I–V contained an extended protein kinase at the C-terminal. Furthermore, most *BnLegLu* genes were lowly expressed and some *BnLegLu* genes were induced by stress, and some *BnLegLus* were particularly induced by *S. sclerotiorum*. Four *BnLegLu* genes were selected based on transcriptional variations in response to SD stimulation and significant sites in GWAS for SD resistance. Furthermore, we experimentally validated that the up-regulated expression of *BnLegLus-16* could inhibit the spread of SD. In addition, we also discussed the possible mechanism for the response of *BnLegLus* to *S. sclerotiorum*, and proposed some directions for further study of *BnLegLus-16*. In summary, this study explores the function of *BnLegLus* in SD resistance, and also provides some clues for further exploration of the role of *BnLegLus* in SD resistance. The results are of great significance for further research on the function of *BnLegLu* gene family for the genetic improvement of agronomic traits or stress tolerance in *B. napus*.

## Data availability statement

The original contributions presented in the study are included in the article/Supplementary Material, and further inquiries can be directed to the corresponding author.

## Author contributions

RZ and ZB: conceptualization and writing—review and editing. RZ and MX: data curation. ZB and SL: funding acquisition. FG: investigation. RZ, MX, JL, and MT: methodology. XC: project administration. RZ: software and writing—original draft. FG and YL: validation. YL: germplasm resources. All authors contributed to the article and approved the submitted version.

## Funding

This research was funded by Central Public-interest Scientific Institution Basal Research Fund, grant numbers 2021-2060302-061-027 and 2021-2060302-061-029; China Agriculture Research System of MOF and MARA (CARS-12); the Agricultural Science and Technology Innovation Program of the Chinese Academy of Agricultural Sciences (CAAS-ASTIP-2013-OCRI); and the National Key Research and Development Program of China grant number U20A2034.

## Conflict of interest

The authors declare that the research was conducted in the absence of any commercial or financial relationships that could be construed as a potential conflict of interest.

## Publisher’s note

All claims expressed in this article are solely those of the authors and do not necessarily represent those of their affiliated organizations, or those of the publisher, the editors and the reviewers. Any product that may be evaluated in this article, or claim that may be made by its manufacturer, is not guaranteed or endorsed by the publisher.

## Footnotes

^1^ https://www.ncbi.nlm.nih.gov/cdd

^2^ https://www.string-db.org/

^3^ http://yanglab.hzau.edu.cn/BnTIR

^4^ http://pfam.xfam.org

^5^ https://www.hmmer.org/

^6^ http://smart.embl.de/

^7^ https://www.ncbi.nlm.nih.gov/cdd2

^8^ https://www.genoscope.cns.fr/brassicanapus/

^9^ https://www.arabidopsis.org/

^10^ http://www.expasy.org/

^11^ https://dtu.biolib.com/DeepTMHMM

^12^ http://cello.life.nctu.edu.tw/

^13^ https://itol.embl.de/

^14^ http://pfam.xfam.org/search

^15^ https://www.ncbi.nlm.nih.gov/cdd

^16^ http://bioinformatics.psb.ugent.be/webtools/plantcare/html/

^17^ https://www.string-db.org/

^18^ https://www.thermofisher.com

^19^ https://www.takarabiomed.com.cn/

^20^ http://www.bio-rad.com/

## References

[ref1] Armijo G. Salinas P. Monteoliva M. I. Seguel A. García C. Villarroel-Candia E.. (2013). A salicylic acid-induced lectin-like protein plays a positive role in the effector-triggered immunity response of *Arabidopsis thaliana* to *Pseudomonas syringae* Avr-Rpm1. Mol. Plant Microbe Interact. 26, 1395–1406. doi: 10.1094/mpmi-02-13-0044-r, PMID: 24006883

[ref2] Arnaud D. Desclos-Theveniau M. Zimmerli L. (2012). Disease resistance to *Pectobacterium carotovorum* is negatively modulated by the Arabidopsis lectin receptor kinase LecRK-V.5. Plant Signal. Behav. 7, 1070–1072. doi: 10.4161/psb.21013, PMID: 22899085 PMC3489629

[ref3] Bailey T. L. Boden M. Buske F. A. Frith M. Grant C. E. Clementi L.. (2009). MEME SUITE: tools for motif discovery and searching. Nucleic Acids Res. 37, W202–W208. doi: 10.1093/nar/gkp335, PMID: 19458158 PMC2703892

[ref4] Bailey P. C. Schudoma C. Jackson W. Baggs E. Dagdas G. Haerty W.. (2018). Dominant integration locus drives continuous diversification of plant immune receptors with exogenous domain fusions. Genome Biol. 19, 23. doi: 10.1186/s13059-018-1392-6, PMID: 29458393 PMC5819176

[ref5] Balagué C. Gouget A. Bouchez O. Souriac C. Haget N. Boutet-Mercey S.. (2017). The *Arabidopsis thaliana* lectin receptor kinase LecRK-I.9 is required for full resistance to pseudomonas syringae and affects jasmonate signalling. Mol. Plant Pathol. 18, 937–948. doi: 10.1111/mpp.12457, PMID: 27399963 PMC6638305

[ref6] Bellande K. Bono J. J. Savelli B. Jamet E. Canut H. (2017). Plant lectins and lectin receptor-like kinases: how do they sense the outside? Int. J. Mol. Sci. 18:1164. doi: 10.3390/ijms18061164, PMID: 28561754 PMC5485988

[ref7] Bouwmeester K. de Sain M. Weide R. Gouget A. Klamer S. Canut H.. (2011). The lectin receptor kinase LecRK-I.9 is a novel Phytophthora resistance component and a potential host target for a RXLR effector. PLoS Pathog. 7:e1001327. doi: 10.1371/journal.ppat.1001327, PMID: 21483488 PMC3068997

[ref8] Cao Y. Yan X. Ran S. Ralph J. Smith R. A. Chen X.. (2022). Knockout of the lignin pathway gene BnF5H decreases the S/G lignin compositional ratio and improves *Sclerotinia sclerotiorum* resistance in *Brassica napus*. Plant Cell Environ. 45, 248–261. doi: 10.1111/pce.14208, PMID: 34697825 PMC9084453

[ref9] Cavada B. S. Osterne V. J. S. Lossio C. F. Pinto-Junior V. R. Oliveira M. V. Silva M. T. L.. (2019). One century of ConA and 40 years of ConBr research: A structural review. Int. J. Biol. Macromol. 134, 901–911. doi: 10.1016/j.ijbiomac.2019.05.100, PMID: 31108148

[ref10] Chalhoub B. Denoeud F. Liu S. Parkin I. A. Tang H. Wang X.. (2014). Plant genetics. Early allopolyploid evolution in the post-Neolithic *Brassica napus* oilseed genome. Science 345, 950–953. doi: 10.1126/science.1253435, PMID: 25146293

[ref11] Chen C. Chen H. Zhang Y. Thomas H. R. Frank M. H. He Y.. (2020). TBtools: An integrative toolkit developed for interactive analyses of big biological data. Mol. Plant 13, 1194–1202. doi: 10.1016/j.molp.2020.06.009, PMID: 32585190

[ref12] Damme E. Lannoo N. Peumans W. J. (2008). Plant Lectins. Adv. Bot. Res. 48, 107–209. doi: 10.1016/S0065-2296(08)00403-5

[ref13] De Coninck T. Van Damme E. J. M. (2021). Review: The multiple roles of plant lectins. Plant Sci. 313:111096. doi: 10.1016/j.plantsci.2021.111096, PMID: 34763880

[ref14] Desclos-Theveniau M. Arnaud D. Huang T. Y. Lin G. J. Chen W. Y. Lin Y. C.. (2012). The Arabidopsis lectin receptor kinase LecRK-V.5 represses stomatal immunity induced by *Pseudomonas syringae* pv. Tomato DC3000. PLoS Pathog. 8:e1002513. doi: 10.1371/journal.ppat.1002513, PMID: 22346749 PMC3276567

[ref15] Ding L. N. Li T. Guo X. J. Li M. Liu X. Y. Cao J.. (2021). Sclerotinia stem rot resistance in rapeseed: recent progress and future prospects. J. Agric. Food Chem. 69, 2965–2978. doi: 10.1021/acs.jafc.0c07351, PMID: 33667087

[ref16] Ding L. N. Li M. Guo X. J. Tang M. Q. Cao J. Wang Z.. (2020). Arabidopsis GDSL1 overexpression enhances rapeseed *Sclerotinia sclerotiorum* resistance and the functional identification of its homolog in *Brassica napus*. Plant Biotechnol. J. 18, 1255–1270. doi: 10.1111/pbi.13289, PMID: 31693306 PMC7152613

[ref17] Edelman G. M. Cunningham B. A. Reeke G. N. Jr. Becker J. W. Waxdal M. J. Wang J. L. (1972). The covalent and three-dimensional structure of concanavalin A. Proc. Natl. Acad. Sci. U. S. A. 69, 2580–2584. doi: 10.1073/pnas.69.9.2580, PMID: 4506778 PMC426993

[ref18] Eggermont L. Verstraeten B. Van Damme E. J. M. (2017). Genome-wide screening for lectin motifs in *Arabidopsis thaliana*. Plant. Genome 10:2. doi: 10.3835/plantgenome2017.02.0010, PMID: 28724081

[ref19] Fedoroff N. V. Battisti D. S. Beachy R. N. Cooper P. J. Fischhoff D. A. Hodges C. N.. (2010). Radically rethinking agriculture for the 21st century. Science 327, 833–834. doi: 10.1126/science.1186834, PMID: 20150494 PMC3137512

[ref20] Fonseca V. J. A. Braga A. L. Filho J. R. Teixeira C. S. da Hora G. C. A. Morais-Braga M. F. B. (2022). A review on the antimicrobial properties of lectins. Int. J. Biol. Macromol. 195, 163–178. doi: 10.1016/j.ijbiomac.2021.11.209, PMID: 34896466

[ref21] Gatehouse A. M. R. Davison G. M. Stewart J. N. Gatehouse L. N. Kumar A. Geoghegan I. E.. (1999). Concanavalin A inhibits development of tomato moth (*Lacanobia oleracea*) and peach-potato aphid (*Myzus persicae*) when expressed in transgenic potato plants. Mol. Breed. 5, 153–165. doi: 10.1023/A:1009681705481

[ref22] Gautam A. K. Gupta N. Narvekar D. T. Bhadkariya R. Bhagyawant S. S. (2018a). Characterization of chickpea (*Cicer arietinum* L.) lectin for biological activity. Physiol. Mol. Biol. Plants 24, 389–397. doi: 10.1007/s12298-018-0508-5, PMID: 29692547 PMC5911256

[ref23] Gautam A. K. Shrivastava N. Sharma B. Bhagyawant S. S. (2018b). Current scenario of legume lectins and their practical applications. J. Crop. Sci. Biotechnol. 21, 217–227. doi: 10.1007/s12892-018-0002-0

[ref24] Girard I. J. Tong C. Becker M. G. Mao X. Huang J. de Kievit T.. (2017). RNA sequencing of *Brassica napus* reveals cellular redox control of Sclerotinia infection. J. Exp. Bot. 68, 5079–5091. doi: 10.1093/jxb/erx338, PMID: 29036633 PMC5853404

[ref25] Hardman K. D. Ainsworth C. F. (1972). Structure of concanavalin A at 2.4-A resolution. Biochemistry 11, 4910–4919. doi: 10.1021/bi00776a006 4638345

[ref26] He X. J. Zhang Z. G. Yan D. Q. Zhang J. S. Chen S. Y. (2004). A salt-responsive receptor-like kinase gene regulated by the ethylene signaling pathway encodes a plasma membrane serine/threonine kinase. Theor. Appl. Genet. 109, 377–383. doi: 10.1007/s00122-004-1641-9, PMID: 15067507

[ref27] Huang P. Y. Yeh Y. H. Liu A. C. Cheng C. P. Zimmerli L. (2014). The Arabidopsis LecRK-VI.2 associates with the pattern-recognition receptor FLS2 and primes Nicotiana benthamiana pattern-triggered immunity. Plant J. 79, 243–255. doi: 10.1111/tpj.12557, PMID: 24844677

[ref28] Jiang S. Y. Ma Z. Ramachandran S. (2010). Evolutionary history and stress regulation of the lectin superfamily in higher plants. BMC Evol. Biol. 10, 79. doi: 10.1186/1471-2148-10-79, PMID: 20236552 PMC2846932

[ref29] Joshi A. Dang H. Q. Vaid N. Tuteja N. (2010). Pea lectin receptor-like kinase promotes high salinity stress tolerance in bacteria and expresses in response to stress in planta. Glycoconj. J. 27, 133–150. doi: 10.1007/s10719-009-9265-6, PMID: 19898933

[ref30] Katoch R. Tripathi A. (2021). Research advances and prospects of legume lectins. J. Biosci. 46:104. doi: 10.1007/s12038-021-00225-8 34815374 PMC8608583

[ref31] Kosová K. Vítámvás P. Prášil I. T. Renaut J. (2011). Plant proteome changes under abiotic stress--contribution of proteomics studies to understanding plant stress response. J. Proteomics 74, 1301–1322. doi: 10.1016/j.jprot.2011.02.006 21329772

[ref32] Kumar S. Stecher G. Tamura K. (2016). MEGA7: molecular evolutionary genetics analysis version 7.0 for bigger datasets. Mol. Biol. Evol. 33, 1870–1874. doi: 10.1093/molbev/msw054, PMID: 27004904 PMC8210823

[ref33] Lagarda-Diaz I. Guzman-Partida A. M. Vazquez-Moreno L. (2017). Legume lectins: proteins with diverse applications. Int. J. Mol. Sci. 18:1242. doi: 10.3390/ijms18061242, PMID: 28604616 PMC5486065

[ref34] Larkin M. A. Blackshields G. Brown N. P. Chenna R. McGettigan P. A. McWilliam H.. (2007). Clustal W and Clustal X version 2.0. Bioinformatics 23, 2947–2948. doi: 10.1093/bioinformatics/btm404, PMID: 17846036

[ref35] Leal A. F. Lopes N. E. Clark A. T. de Pontes Filho N. T. Beltrão E. I. Neves R. P. (2012). Carbohydrate profiling of fungal cell wall surface glycoconjugates of Aspergillus species in brain and lung tissues using lectin histochemistry. Med. Mycol. 50, 756–759. doi: 10.3109/13693786.2011.631946, PMID: 22103341

[ref36] Li Z. Chakraborty S. Xu G. (2016). X-ray crystallographic studies of the extracellular domain of the first plant ATP receptor, DORN1, and the orthologous protein from *Camelina sativa*. Acta Crystallogr. F Struct. Biol. Commun. 72, 782–787. doi: 10.1107/s2053230x16014278, PMID: 27710944 PMC5053164

[ref37] Li T. Yin X. Liu D. Ma X. Lv H. Sun S. (2012). Isolation and characterization of a novel lectin with antifungal and antiproliferative activities from Sophora alopecuroides seeds. Acta Biochim. Biophys. Sin. Shanghai 44, 606–613. doi: 10.1093/abbs/gms037, PMID: 22634632

[ref38] Liang X. Rollins J. A. (2018). Mechanisms of broad host range necrotrophic pathogenesis in *Sclerotinia sclerotiorum*. Phytopathology 108, 1128–1140. doi: 10.1094/phyto-06-18-0197-rvw, PMID: 30048598

[ref39] Liu F. Li X. Wang M. Wen J. Yi B. Shen J.. (2018). Interactions of WRKY15 and WRKY33 transcription factors and their roles in the resistance of oilseed rape to Sclerotinia infection. Plant Biotechnol. J. 16, 911–925. doi: 10.1111/pbi.12838, PMID: 28929638 PMC5867032

[ref40] Liu D. Wu J. Lin L. Li P. Li S. Wang Y.. (2021a). Overexpression of Cinnamoyl-CoA reductase 2 in *Brassica napus* increases resistance to *Sclerotinia sclerotiorum* by affecting lignin biosynthesis. Front. Plant Sci. 12:732733. doi: 10.3389/fpls.2021.732733, PMID: 34630482 PMC8494948

[ref41] Liu D. Yu L. Wei L. Yu P. Wang J. Zhao H.. (2021b). BnTIR: an online transcriptome platform for exploring RNA-seq libraries for oil crop *Brassica napus*. Plant Biotechnol. J. 19, 1895–1897. doi: 10.1111/pbi.13665, PMID: 34260132 PMC8486221

[ref42] Livak K. J. Schmittgen T. D. (2001). Analysis of relative gene expression data using real-time quantitative PCR and the 2(-Delta Delta C(T)) method. Methods 25, 402–408. doi: 10.1006/meth.2001.1262 11846609

[ref43] Lu S. Wang J. Chitsaz F. Derbyshire M. K. Geer R. C. Gonzales N. R.. (2020). CDD/SPARCLE: the conserved domain database in 2020. Nucleic Acids Res. 48, D265–d268. doi: 10.1093/nar/gkz991, PMID: 31777944 PMC6943070

[ref44] Luo X. Xu N. Huang J. Gao F. Zou H. Boudsocq M.. (2017). A lectin receptor-like kinase mediates pattern-triggered salicylic acid signaling. Plant Physiol. 174, 2501–2514. doi: 10.1104/pp.17.00404, PMID: 28696275 PMC5543950

[ref45] Lyou S. H. Park H. J. Jung C. Sohn H. B. Lee G. Kim C. H.. (2009). The Arabidopsis AtLEC gene encoding a lectin-like protein is up-regulated by multiple stimuli including developmental signal, wounding, jasmonate, ethylene, and chitin elicitor. Mol. Cells 27, 75–81. doi: 10.1007/s10059-009-0007-1, PMID: 19214436

[ref46] Manoj N. Suguna K. (2001). Signature of quaternary structure in the sequences of legume lectins. Protein Eng. 14, 735–745. doi: 10.1093/protein/14.10.735, PMID: 11739891

[ref47] Mistry J. Chuguransky S. Williams L. Qureshi M. Salazar G. A. Sonnhammer E. L. L.. (2021). Pfam: The protein families database in 2021. Nucleic Acids Res. 49, D412–d419. doi: 10.1093/nar/gkaa913, PMID: 33125078 PMC7779014

[ref48] Myers R. J. Fichman Y. Stacey G. Mittler R. (2022). Extracellular ATP plays an important role in systemic wound response activation. Plant Physiol. 189, 1314–1325. doi: 10.1093/plphys/kiac148, PMID: 35348752 PMC9237675

[ref49] Nivetha R. Meenakumari M. Bhuvaragavan S. Hilda K. Janarthanan S. (2021). In silico analysis of carbohydrate-binding pockets in the lectin genes from various species of Canavalia. Comput. Biol. Chem. 92:107477. doi: 10.1016/j.compbiolchem.2021.107477, PMID: 33773472

[ref50] Nováková M. Sašek V. Dobrev P. I. Valentová O. Burketová L. (2014). Plant hormones in defense response of *Brassica napus* to Sclerotinia sclerotiorum—reassessing the role of salicylic acid in the interaction with a necrotroph. Plant Physiol. Biochem. 80, 308–317. doi: 10.1016/j.plaphy.2014.04.019, PMID: 24837830

[ref51] Passricha N. Saifi S. K. Kharb P. Tuteja N. (2019). Marker-free transgenic rice plant overexpressing pea LecRLK imparts salinity tolerance by inhibiting sodium accumulation. Plant Mol. Biol. 99, 265–281. doi: 10.1007/s11103-018-0816-8, PMID: 30604324

[ref52] Pham A. Q. Cho S. H. Nguyen C. T. Stacey G. (2020). Arabidopsis Lectin receptor kinase P2K2 is a second plant receptor for extracellular ATP and contributes to innate immunity. Plant Physiol. 183, 1364–1375. doi: 10.1104/pp.19.01265, PMID: 32345768 PMC7333714

[ref53] Saijo Y. Loo E. P. (2020). Plant immunity in signal integration between biotic and abiotic stress responses. New Phytol. 225, 87–104. doi: 10.1111/nph.15989, PMID: 31209880

[ref54] Shannon P. Markiel A. Ozier O. Baliga N. S. Wang J. T. Ramage D.. (2003). Cytoscape: a software environment for integrated models of biomolecular interaction networks. Genome Res. 13, 2498–2504. doi: 10.1101/gr.1239303, PMID: 14597658 PMC403769

[ref55] Singh N. K. Goodman A. Walter P. Helms V. Hayat S. (2011). TMBHMM: a frequency profile based HMM for predicting the topology of transmembrane beta barrel proteins and the exposure status of transmembrane residues. Biochim. Biophys. Acta 1814, 664–670. doi: 10.1016/j.bbapap.2011.03.004, PMID: 21426944

[ref56] Singh P. Zimmerli L. (2013). Lectin receptor kinases in plant innate immunity. Front. Plant Sci. 4:124. doi: 10.3389/fpls.2013.00124, PMID: 23675375 PMC3646242

[ref57] Sitohy M. Doheim M. Badr H. (2007). Isolation and characterization of a lectin with antifungal activity from Egyptian *Pisum sativum* seeds. Food Chem. 104, 971–979. doi: 10.1016/j.foodchem

[ref58] Sparkes I. A. Runions J. Kearns A. Hawes C. (2006). Rapid, transient expression of fluorescent fusion proteins in tobacco plants and generation of stably transformed plants. Nat. Protoc. 1, 2019–2025. doi: 10.1038/nprot.2006.286, PMID: 17487191

[ref59] Sprawka I. Goławska S. Parzych T. Sytykiewicz H. Czerniewicz P. (2015). Apoptosis induction by concanavalin A in gut cells of grain aphid. Arthropod. Plant Interact. 9, 133–140. doi: 10.1007/s11829-015-9356-1

[ref60] Sun Z. Zang Y. Zhou L. Song Y. Chen D. Zhang Q.. (2021). A tomato receptor-like cytoplasmic kinase, SlZRK1, acts as a negative regulator in wound-induced jasmonic acid accumulation and insect resistance. J. Exp. Bot. 72, 7285–7300. doi: 10.1093/jxb/erab350, PMID: 34309647

[ref61] Szklarczyk D. Gable A. L. Lyon D. Junge A. Wyder S. Huerta-Cepas J.. (2019). STRING v11: protein-protein association networks with increased coverage, supporting functional discovery in genome-wide experimental datasets. Nucleic Acids Res. 47, D607–d613. doi: 10.1093/nar/gky1131, PMID: 30476243 PMC6323986

[ref62] Tang M.Q. (2019). Population genome variations and subgenome asymmetry in *Brassica napus* L. Huazhong Agricultural University Wuhan, China, D50–D59.

[ref63] Tsaneva M. Van Damme E. J. M. (2020). 130 years of plant Lectin research. Glycoconj. J. 37, 533–551. doi: 10.1007/s10719-020-09942-y, PMID: 32860551 PMC7455784

[ref64] Van Damme E. J. M. (2022). 35 years in plant lectin research: a journey from basic science to applications in agriculture and medicine. Glycoconj. J. 39, 83–97. doi: 10.1007/s10719-021-10015-x, PMID: 34427812 PMC8383723

[ref65] Van Holle S. Van Damme E. J. (2015). Distribution and evolution of the lectin family in soybean (*Glycine max*). Molecules 20, 2868–2891. doi: 10.3390/molecules20022868, PMID: 25679048 PMC6272470

[ref66] Wang Y. Cordewener J. H. America A. H. Shan W. Bouwmeester K. Govers F. (2015). Arabidopsis lectin receptor kinases LecRK-IX.1 and LecRK-IX.2 are functional analogs in regulating phytophthora resistance and plant cell death. Mol. Plant Microbe Interact. 28, 1032–1048. doi: 10.1094/mpmi-02-15-0025-r, PMID: 26011556

[ref67] Wang C. Huang X. Li Q. Zhang Y. Li J. L. Mou Z. (2019). Extracellular pyridine nucleotides trigger plant systemic immunity through a lectin receptor kinase/BAK1 complex. Nat. Commun. 10, 4810. doi: 10.1038/s41467-019-12781-7, PMID: 31641112 PMC6805918

[ref68] Wang Y. Nsibo D. L. Juhar H. M. Govers F. Bouwmeester K. (2016). Ectopic expression of Arabidopsis L-type lectin receptor kinase genes LecRK-I.9 and LecRK-IX.1 in Nicotiana benthamiana confers Phytophthora resistance. Plant Cell Rep. 35, 845–855. doi: 10.1007/s00299-015-1926-2, PMID: 26795144 PMC4799253

[ref69] Wang Z. Wan L. Xin Q. Chen Y. Zhang X. Dong F.. (2018a). Overexpression of OsPGIP2 confers Sclerotinia sclerotiorum resistance in *Brassica napus* through increased activation of defense mechanisms. J. Exp. Bot. 69, 3141–3155. doi: 10.1093/jxb/ery138, PMID: 29648614 PMC5972623

[ref70] Wang L. Wilkins K. A. Davies J. M. (2018b). Arabidopsis DORN1 extracellular ATP receptor; activation of plasma membrane K(+) -and Ca(2+) -permeable conductances. New Phytol. 218, 1301–1304. doi: 10.1111/nph.15111, PMID: 29574778

[ref71] Wang C. Zhou M. Zhang X. Yao J. Zhang Y. Mou Z. (2017). A lectin receptor kinase as a potential sensor for extracellular nicotinamide adenine dinucleotide in *Arabidopsis thaliana*. Elife 6:e25474. doi: 10.7554/eLife.25474, PMID: 28722654 PMC5560858

[ref72] Wu J. Zhao Q. Liu S. Shahid M. Lan L. Cai G.. (2016). Genome-wide association study identifies new loci for resistance to Sclerotinia stem rot in *Brassica napus*. Front. Plant Sci. 7:1418. doi: 10.3389/fpls.2016.01418, PMID: 27703464 PMC5028409

[ref73] Xin Z. Wang A. Yang G. Gao P. Zheng Z. L. (2009). The Arabidopsis A4 subfamily of lectin receptor kinases negatively regulates abscisic acid response in seed germination. Plant Physiol. 149, 434–444. doi: 10.1104/pp.108.130583, PMID: 18987212 PMC2613733

[ref74] Xu N. Luo X. Wu W. Xing Y. Liang Y. Liu Y.. (2020). A plant Lectin receptor-like kinase phosphorylates the bacterial effector AvrPtoB to dampen its virulence in Arabidopsis. Mol. Plant 13, 1499–1512. doi: 10.1016/j.molp.2020.09.016, PMID: 32977056

[ref75] Yekondi S. Liang F. C. Okuma E. Radziejwoski A. Mai H. W. Swain S.. (2018). Nonredundant functions of Arabidopsis LecRK-V.2 and LecRK-VII.1 in controlling stomatal immunity and jasmonate-mediated stomatal closure. New Phytol. 218, 253–268. doi: 10.1111/nph.14953, PMID: 29250804

[ref76] Yu C. S. Lin C. J. Hwang J. K. (2004). Predicting subcellular localization of proteins for gram-negative bacteria by support vector machines based on n-peptide compositions. Protein Sci. 13, 1402–1406. doi: 10.1110/ps.03479604, PMID: 15096640 PMC2286765

[ref77] Yu G. Wang L. G. Han Y. He Q. Y. (2012). clusterProfiler: an R package for comparing biological themes among gene clusters. OMICS 16, 284–287. doi: 10.1089/omi.2011.0118, PMID: 22455463 PMC3339379

[ref78] Zhang Y. Ali U. Zhang G. Yu L. Fang S. Iqbal S.. (2019). Transcriptome analysis reveals genes commonly responding to multiple abiotic stresses in rapeseed. Mol. Breed. 39, 158. doi: 10.1007/s11032-019-1052-x

[ref79] Zhang Y. Fang Q. Zheng J. Li Z. Li Y. Feng Y.. (2022). GmLecRlk, a Lectin receptor-like protein kinase, contributes to salt stress tolerance by regulating salt-responsive genes in soybean. Int. J. Mol. Sci. 23:1030. doi: 10.3390/ijms23031030, PMID: 35162952 PMC8835537

