# Supplementary material for: Genome-wide identification and functional exploration of the legume lectin genes in Brassica napus and their roles in Sclerotinia disease resistance
Source: Front Plant Sci. 2022 Jul 22;13:963263. doi: 10.3389/fpls.2022.963263 (PMC9374194; doi:10.3389/fpls.2022.963263)
Supplement: Supplementary file 1 [file Data_Sheet_1.PDF]

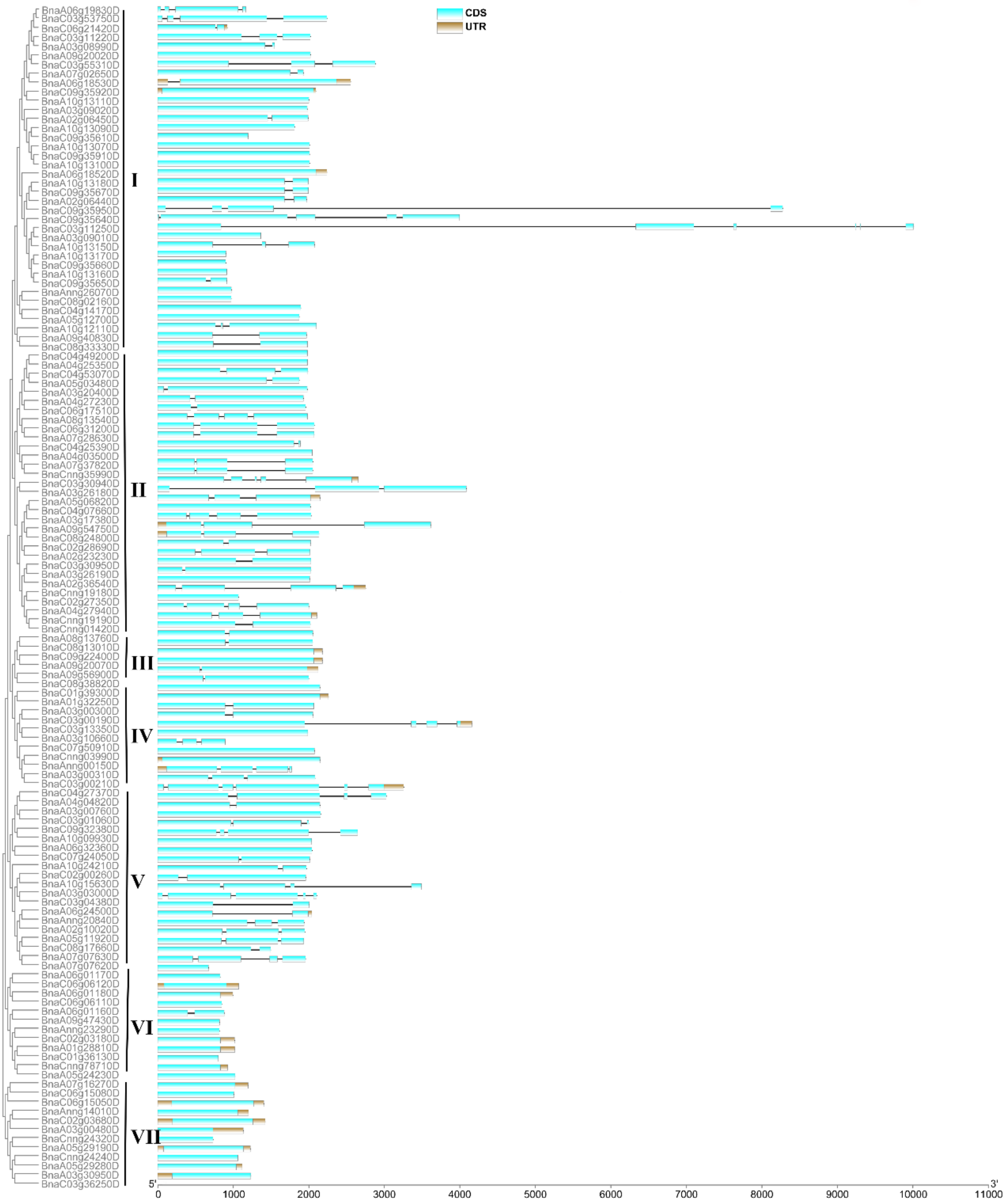

Figure S1. The phylogenetic relationship and gene structure of *BnLegLus* genes in *B. napus*. (a) The phylogenetic relationship of BnLegLu proteins (b) Gene structures of the *BnLegLu* genes. Blue boxes represented the CDS, whereas, orange boxes represented UTR.

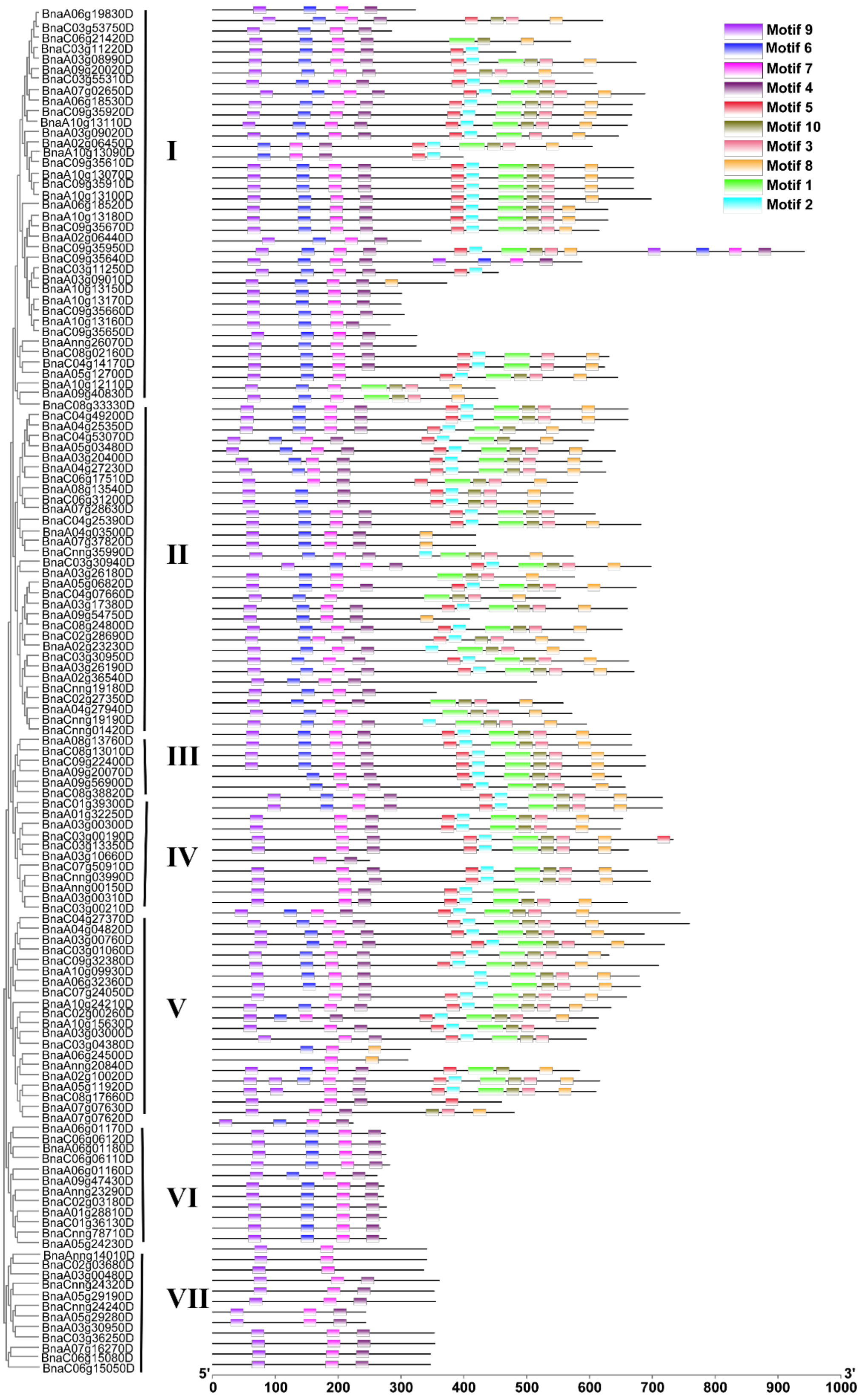

Figure S2 The phylogenetic relationship and conserved motifs of BnLegLus in *B. napus*. (a) The phylogenetic relationship of BnLegLu proteins (b) The motif composition of BnLegLu proteins. Motifs (1–10) were shown in different colored boxes.

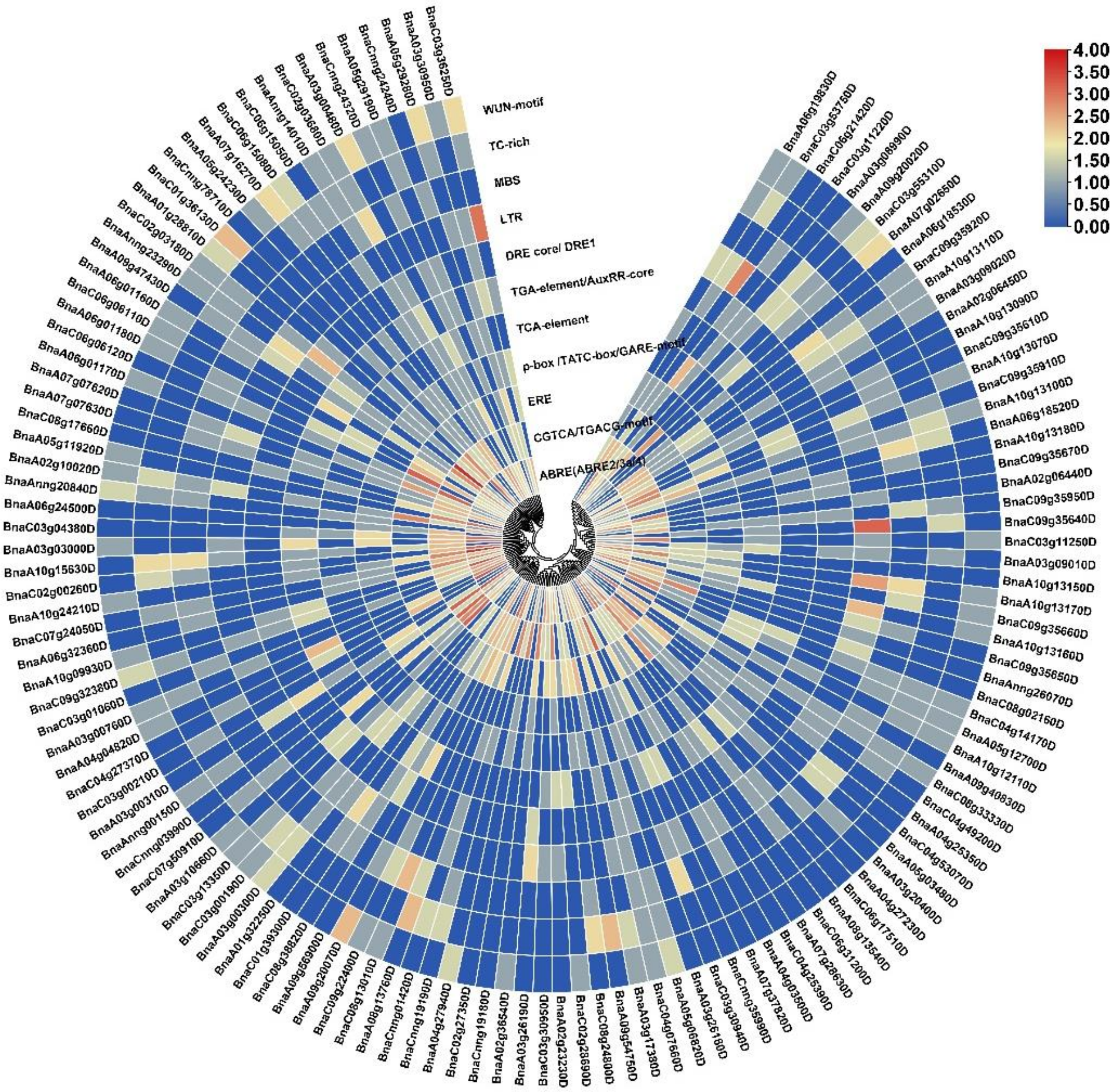

Figure S3. Cis-acting regulatory elements identified in *BnLegLus* promoters in *B. napus*. The color code shows log2 of copy number of cis-elements from low (blue) to high (red).

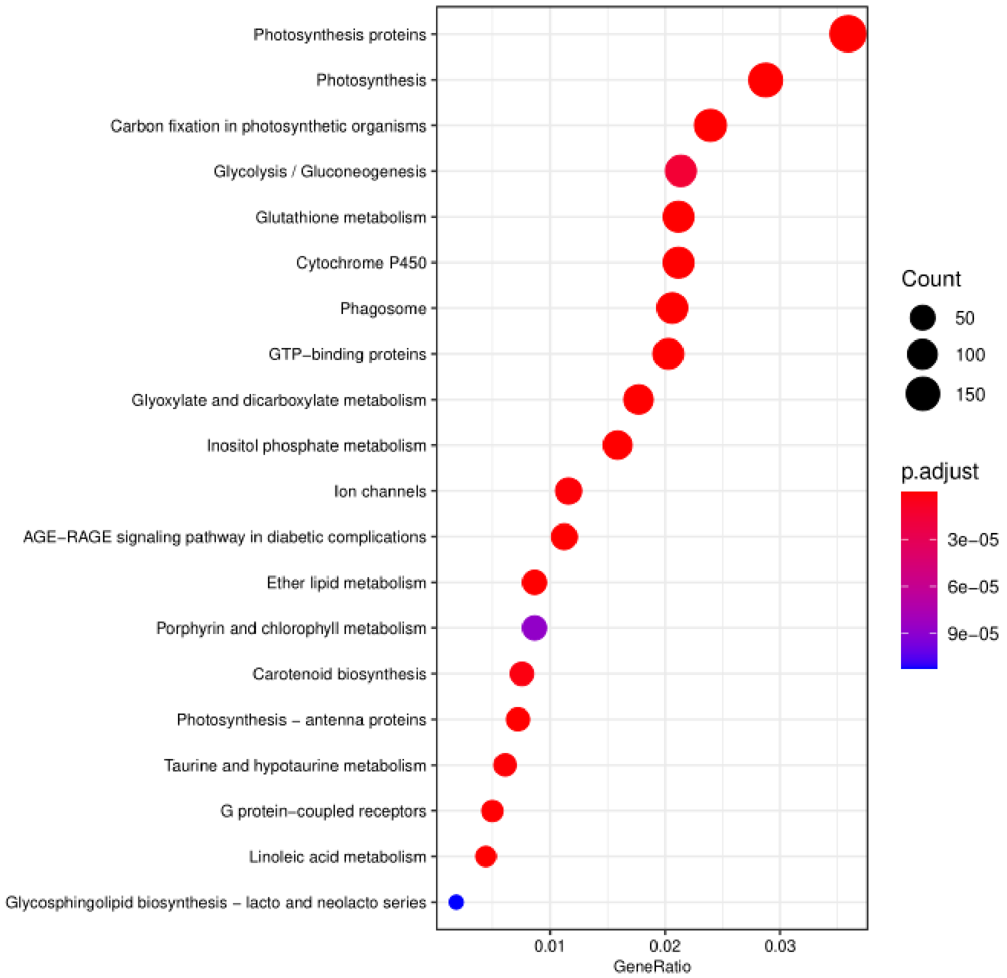

Figure S4. KEGG pathway analysis of proteins interacted with BnLegLu proteins.

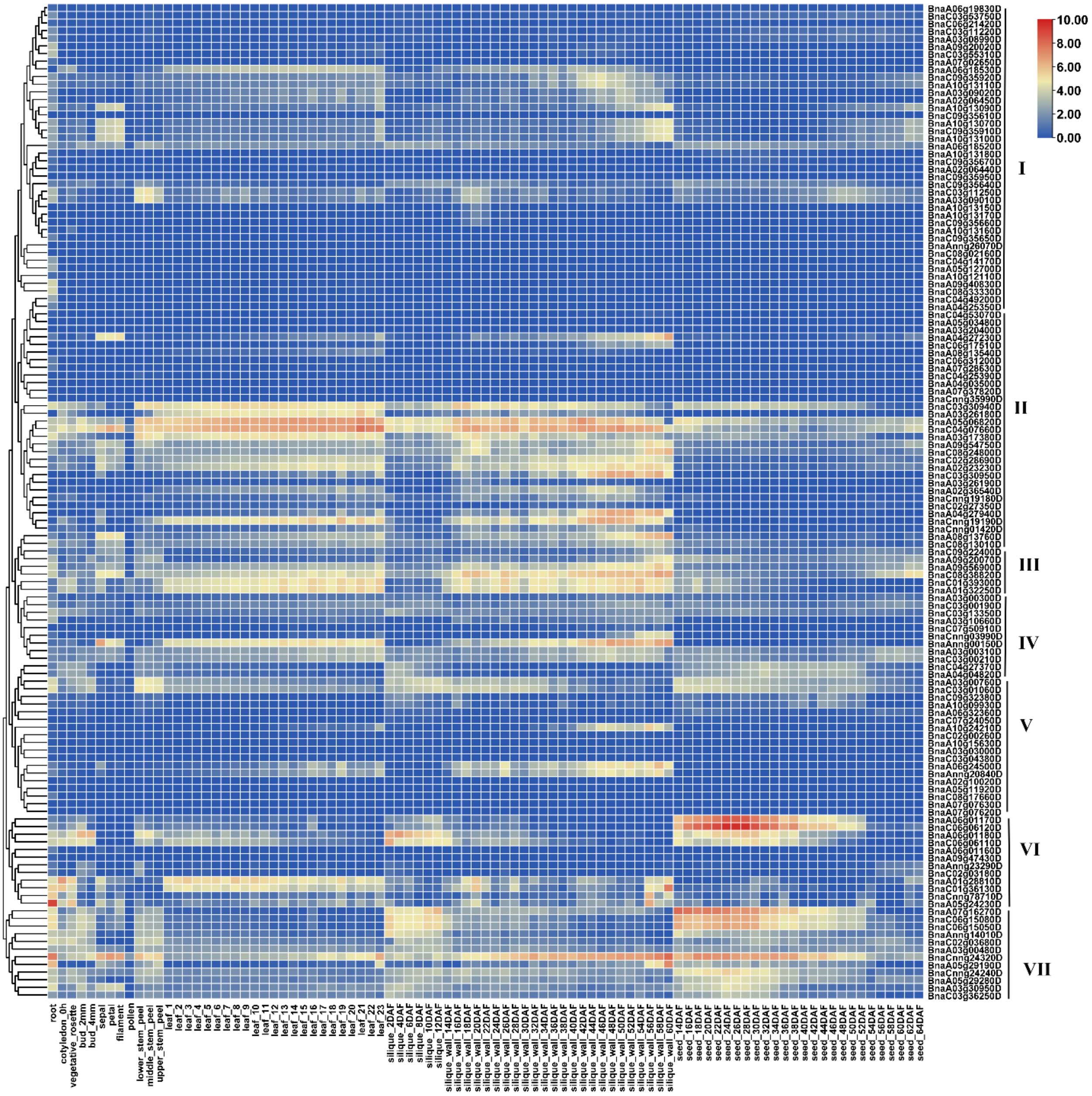

Figure S5. Expression profile of *BnLegLus* in different tissues of *B. napus*. Heatmap was generated by taking log2 fold of FPKM values. The color bar showed relative expression from low (blue) to high (red).

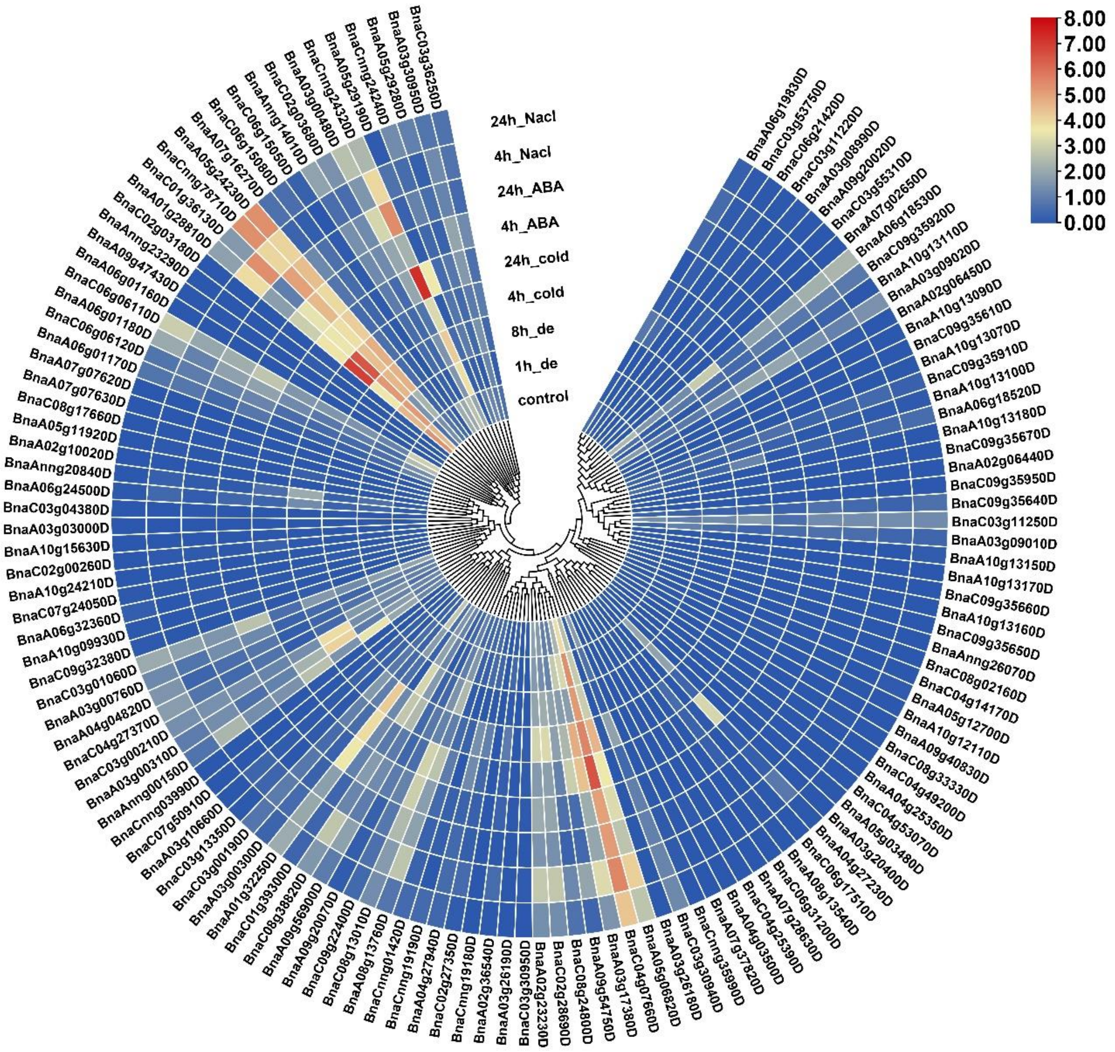

Figure S6. Expression profile of *BnLegLu* genes under abiotic stress conditions in *B. napus*. The expression data was processed by comparing the control with each treated sample and calculated the log2 fold change to generate the Heatmap. The color scale represented relative expression levels from low (blue) to high (red).

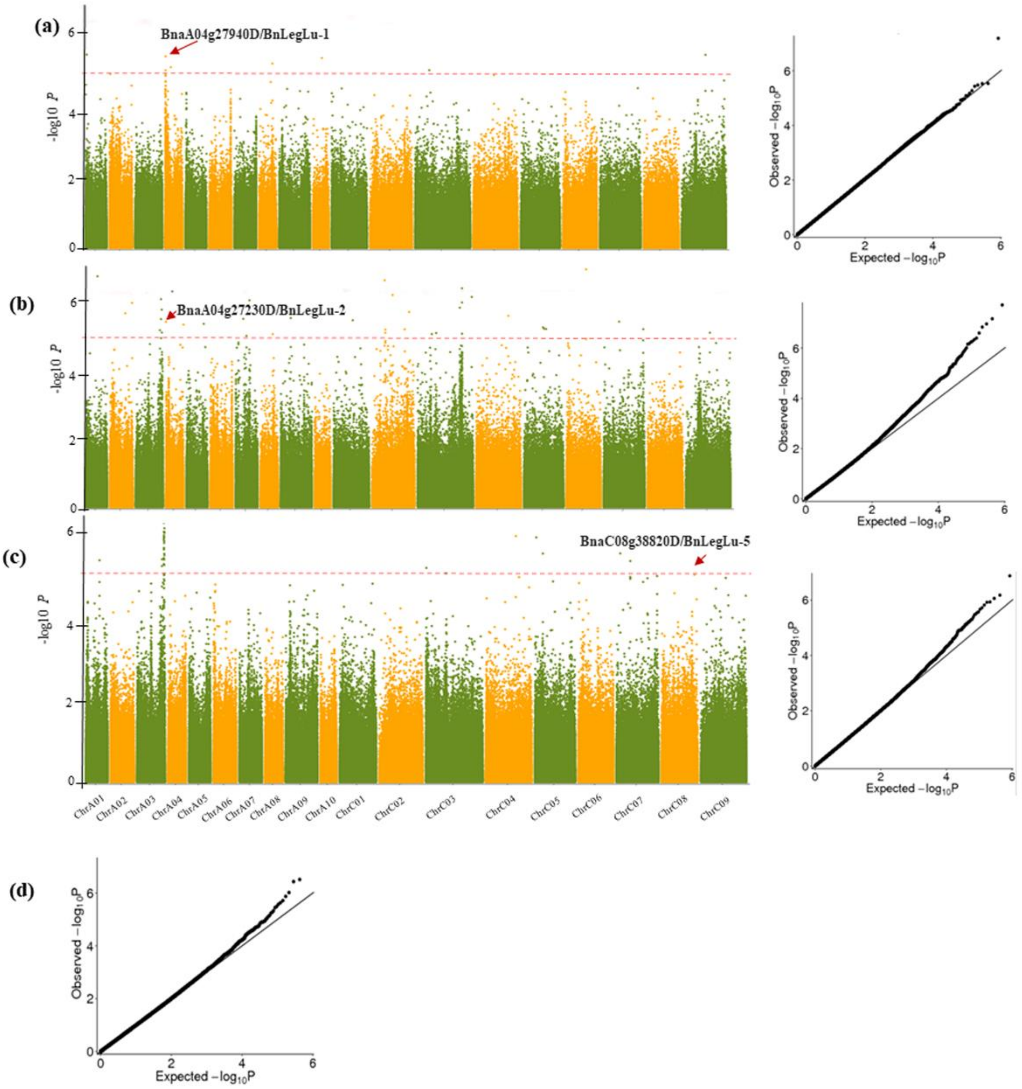

Figure S7 The genome-wide association analysis (GWAS) for SD resistance in a *B. napus* population comprising 324 accessions and Manhattan plots of SD resistance from association analyses: (a) Manhattan plots of the disease after 48 h–24 h_all; (b) Manhattan plots of the disease after 48 h-24 h_1; (c) Manhattan plots of the disease after 48 h-36 h_1; (d) QQ plot (Quantile-Quantile plots) for GWAS of the disease_36h-all. The red dashed line shows GWAS threshold (1/SNP number).
